# Exploring Lipid Metabolic Reprogramming: Mechanistic Insights and Implications for Tumor Radiotherapy

**DOI:** 10.7150/ijbs.133227

**Published:** 2026-06-17

**Authors:** Ruiqiu Zhu, Ying Wan, Jinrong Wei, Limin Jin, Yuntian Shen, Yaqun Zhu, Qiliang Peng

**Affiliations:** 1 Department of Radiotherapy & Oncology, The Second Affiliated Hospital of Soochow University, Suzhou, China.; 2 Department of Pharmacy, Zhongshan Hospital, Fudan University, Shanghai, China.; 3 Department of General Surgery, The Second Affiliated Hospital of Soochow University, Suzhou, China.

**Keywords:** lipid metabolism, metabolic reprogramming, radioresistance, tumor microenvironment

## Abstract

Lipid metabolic reprogramming plays a crucial role in modulating tumor responses to radiotherapy by influencing radiation-induced oxidative damage, membrane repair, ferroptosis, energy stress, and immune regulation. Within the context of ionizing radiation, lipid pathways of particular significance include iron-dependent lipid peroxidation and ferroptosis, cholesterol and phospholipid remodeling that impacts membrane integrity and lipid rafts, lipid droplet-mediated buffering of metabolic stress, fatty acid oxidation-dependent energy supply, and sphingolipid-regulated apoptosis. This review delineates pre-existing tumor lipid programs from IR-induced adaptive responses, highlighting that their contributions to radiosensitivity or radioresistance are contingent upon tumor lineage, genetic background, microenvironmental conditions, and treatment context. The coupling of cancer cells with their microenvironment through lipid interactions, encompassing intercellular lipid transfer, nutrient competition, paracrine lipid mediators, and exosome-mediated signaling, is identified as a central component of radioresistance. In conclusion, therapeutic opportunities are evaluated based on their translational maturity, encompassing a spectrum from mechanistic concepts and preclinical radiosensitization strategies to approaches with emerging clinical significance. This synthesis, focused on radiotherapy, seeks to elucidate how lipid vulnerabilities can be strategically and judiciously exploited to enhance radiation outcomes.

## Introduction

Tumor metabolic remodeling is intricately linked to malignant progression and therapeutic resistance 1,2. Within the spectrum of these metabolic alterations, lipid reprogramming holds particular significance for radiotherapy, as numerous radiation-induced biological effects converge on lipid-dependent pathways. Ionizing radiation induces the formation of reactive oxygen species (ROS), which trigger lipid peroxidation and ferroptosis, while simultaneously exerting stress on cellular membranes, organelles, energy metabolism, and antioxidant defense mechanisms. In response to these challenges, tumor cells may enhance lipid synthesis, fatty acid oxidation, cholesterol remodeling, lipid droplet dynamics, and sphingolipid signaling to facilitate membrane repair, the DNA damage response, metabolic adaptation, and post-irradiation survival. Concurrently, lipid metabolism within the irradiated tumor microenvironment (TME) affects immune suppression, stromal support, and intercellular metabolic communication. These interrelated processes underscore the importance of investigating lipid metabolic reprogramming as a critical factor influencing tumor radiosensitivity and radioresistance.

Radiotherapy represents a pivotal approach in tumor treatment, with its efficacy primarily dependent on the disruption of essential biological processes within tumor cells. Recent studies have demonstrated that ionizing radiation (IR) not only causes direct DNA damage but also significantly perturbs lipid homeostasis in tumor cells, thus serving as a key mechanism of cellular lethality 3,4. IR-induced ROS preferentially target membrane phospholipids (PLs) rich in polyunsaturated fatty acids (PUFAs), triggering a cascade of iron-dependent lipid peroxidation. In conjunction with the impairment of the antioxidant defense system by IR, lipid peroxides accumulate irreversibly, ultimately leading to non-apoptotic ferroptosis and the disintegration of membrane structures 5,6. Additionally, IR exerts a synergistic cytotoxic effect on tumor cells through various pathways, including the destabilization of membranes via disruption of cholesterol/phospholipid homeostasis, depletion of lipid droplets (LDs) energy reserves, inhibition of FAO for energy production, and reprogramming of sphingolipid metabolism 7-11. This mechanistic insight into the disruption of lipid networks by IR establishes a rational basis for the development of innovative therapeutic strategies aimed at improving the efficacy of radiotherapy. For example, leveraging the susceptibility of tumors to ferroptosis induced by IR-generated ROS has prompted investigations into the combination of radiotherapy with ferroptosis inducers, or agents that enhance lipid peroxidation. Additionally, targeting the compensatory upregulation of enzymes such as CPT1A or FASN, which is observed following radiation exposure, provides a direct approach to counteract acquired metabolic resistance. A comprehensive understanding of the specific lipid metabolic dependencies and adaptations of tumors enables radiation oncologists to devise precision interventions that either synergistically enhance the cytotoxic effects of IR or circumvent resistance mechanisms.

The intrinsic reprogramming of lipid metabolism in tumors, coupled with dynamic metabolic adaptations in response to radiotherapy-induced stress, constitutes a significant obstacle in overcoming radioresistance. Tumor cells can develop primary resistance by sustaining heightened activities in fatty acid synthesis, fatty acid oxidation (FAO, β-oxidation), cholesterol synthesis, and LDs accumulation 12. Radiotherapy exacerbates this resistance by inducing the upregulation of enzymes such as carnitine palmitoyltransferase 1A (CPT1A) and fatty acid synthase (FASN)/ peroxisome proliferator-activated receptor-gamma coactivator 1-alpha (PGC1α) 13-15, which in turn reconstruct membrane phospholipid and cholesterol metabolism to facilitate acquired adaptation 16-18. Furthermore, the interactions of lipid metabolism within the TME add an additional layer of complexity to this process. For example, the uptake of lipids by tumor-associated macrophages (TAMs) can promote M2 polarization. Concurrently, the lipotoxic exhaustion of CD8⁺ T cells, the fatty acid reliance of regulatory T cells (Tregs), lipid provision by cancer-associated fibroblasts (CAFs), and exosome-mediated transmission of metabolic reprogramming signals collectively contribute to the establishment of an immunosuppressive TME, thereby reducing the effectiveness of radiotherapy 19-24. A key mechanism of resistance involves tumor cells circumventing radiation-induced damage by enhancing ferroptosis defense mechanisms and demonstrating plasticity in metabolic pathways, including those involving glucose, lipids, and glutamine.

**Figure 1:**
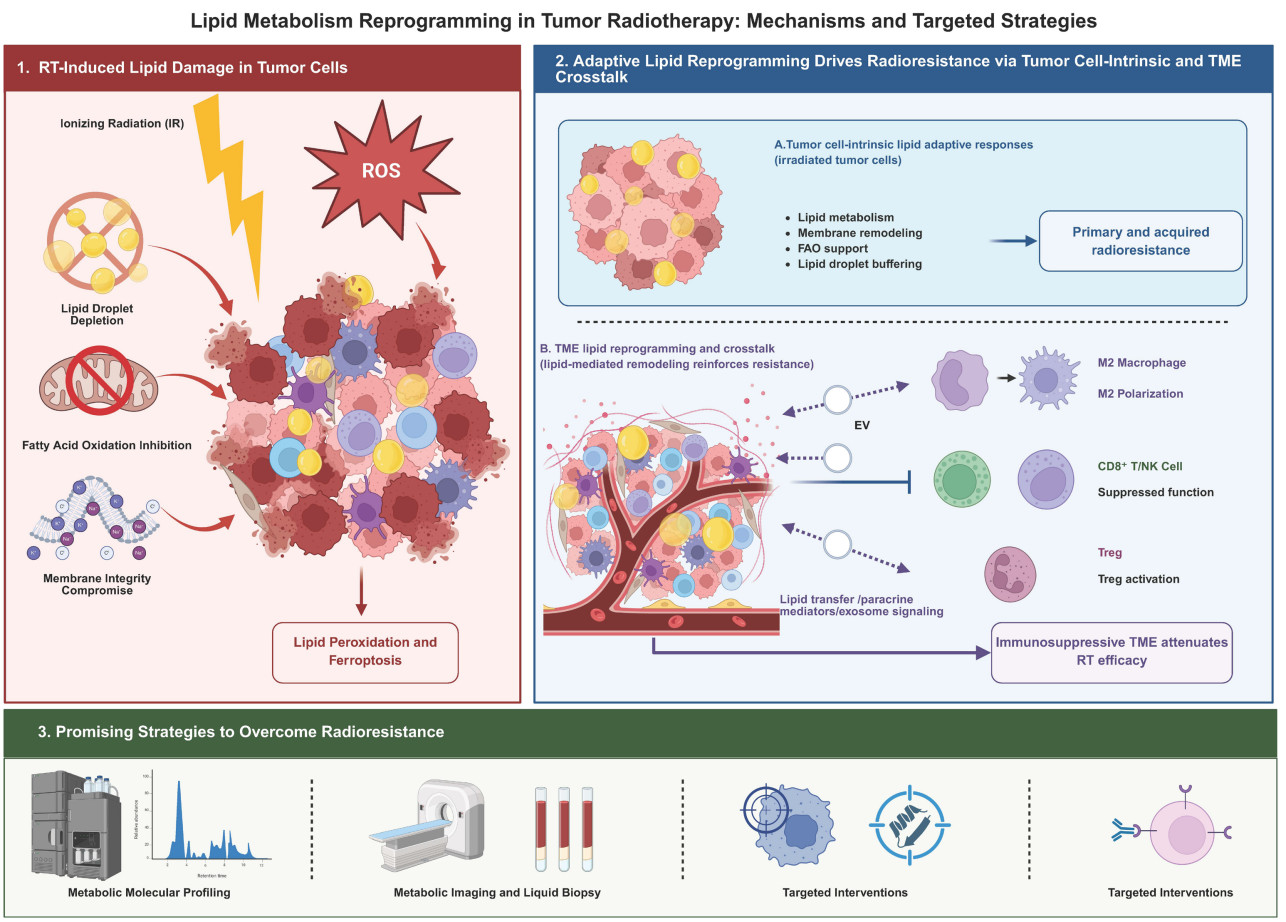
** Schematic overview of lipid metabolic reprogramming in tumor radiotherapy and its therapeutic implications.** Ionizing radiation induces lipid-dependent damage in tumor cells through ROS generation, lipid peroxidation, ferroptotic pressure, membrane disruption, lipid droplet depletion, and impaired fatty acid oxidation. In response, tumor cells activate intrinsic lipid adaptive programs, including enhanced lipid metabolism, membrane remodeling, FAO support, and lipid droplet buffering, which contribute to primary and acquired radioresistance. In parallel, lipid-mediated crosstalk within the tumor microenvironment, involving macrophage polarization, Treg activation, suppression of CD8⁺ T/NK cells, and stromal support, reinforces an immunosuppressive niche and attenuates radiotherapy efficacy. Targeting these tumor-intrinsic and TME-associated lipid vulnerabilities through metabolic profiling, imaging, liquid biopsy, and lipid-directed interventions may provide strategies to overcome radioresistance.

Building upon this rationale, this review initially delineates the principal lipid metabolic pathways that influence tumor responses to irradiation, encompassing fatty acid metabolism, lipid droplet dynamics, phospholipid and cholesterol remodeling, and sphingolipid signaling. Subsequently, it examines the disruption of lipid homeostasis by IR and how tumor cells exploit existing lipid pathways or radiation-induced adaptive mechanisms to withstand oxidative stress, membrane damage, energy stress, and ferroptotic pressure. Special emphasis is placed on the context-dependent nature of these mechanisms across various tumor types, genetic backgrounds, microenvironmental conditions, and treatment settings. Furthermore, the review integrates the coupling of cancer cell and TME lipid metabolism into the discourse on radioresistance, emphasizing lipid transfer, nutrient competition, paracrine lipid mediators, and exosome-mediated communication among tumor, immune, and stromal compartments. In conclusion, lipid-targeted strategies are assessed based on their mechanistic rationale, preclinical evidence of radiosensitization, and emerging clinical significance. The graphical abstract is shown in **Figure 1**.

## Fundamental Attributes of Lipid Metabolic Reprogramming in Tumor Cells

**Figure 2:**
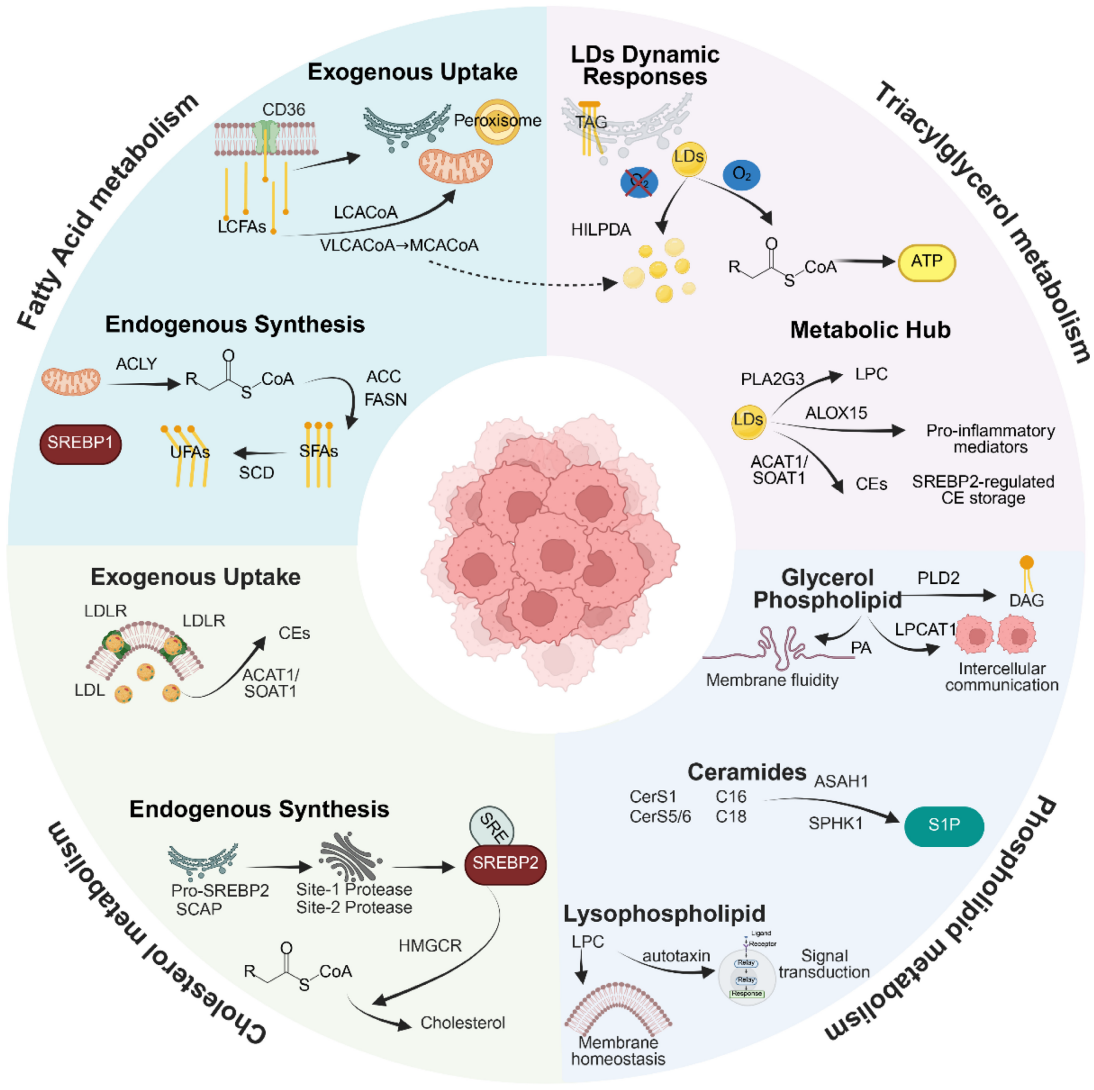
**Architecture of Lipid Metabolic Reprogramming in Malignant Tumors.** This graphical abstract delineates the interconnected framework of tumor lipid metabolic reprogramming, organized into four principal domains. The domain of Fatty Acid Metabolism is characterized by endogenous synthesis through the ACLY/ACC/FASN enzymatic cascade, which is under the transcriptional control of SREBP1. This process is complemented by exogenous uptake mediated by CD36/FABP transporters and activation dependent on ACSL, directing acyl-CoA towards mitochondrial FAO or peroxisomal chain-shortening. The domain of Triglyceride Metabolism illustrates the dynamics responsive to hypoxia or reoxygenation, governed by HIF-1α or HILPDA-mediated suppression of ATGL and phosphorylation-dependent lipolysis of PLIN2. Additionally, LDs exhibit multifunctionality in generating pro-inflammatory lipid mediators (ALOX15/PLA2G3) and storing CEs synthesized by ACAT1/SOAT1. The domain of Phospholipid Metabolism emphasizes glycerophospholipid remodeling through LPCAT1-driven saturation of phosphatidylcholine, which modulates membrane fluidity. It also involves the generation of phosphatidic acid and DAG signaling precursors by PLD2, and the regulation of sphingolipid balance through CerS1/5/6-mediated ceramide synthesis with chain length specificity, alongside SPHK1-driven S1P signaling via SPNS2 export. Finally, the domain of Cholesterol Metabolism features *de novo* synthesis regulated by SREBP2, LDLR-mediated exogenous uptake. Created with BioRender.com.

Lipid metabolic reprogramming is a defining characteristic of malignant tumors and is evident across a wide range of cancer types. Lipids serve as essential structural components of biological membranes and function as energy storage molecules, signaling mediators, and regulators of membrane protein activity 1,25,26. These properties make lipid metabolism particularly relevant to radiotherapy, as radiation-induced oxidative stress, membrane injury, ferroptosis, and metabolic stress are all closely linked to lipid composition and lipid flux. Tumor cells often exhibit increased dependence on lipid acquisition, synthesis, storage, and remodeling compared with normal tissues. The major lipid processes involved include fatty acid uptake and synthesis, triglyceride storage and hydrolysis within lipid droplets, phospholipid and sphingolipid remodeling, and regulation of cholesterol homeostasis 27-32. Together, these interconnected modules provide the structural, energetic, and signaling basis through which tumor cells may respond to, adapt to, or resist ionizing radiation. This metabolic network architecture is depicted in **Figure 2**.

### Fatty acid metabolic reprogramming

Tumor cells reconfigure fatty acid metabolism through the orchestrated activation of endogenous synthesis, exogenous uptake, and catabolic pathways to meet the requirements for biomembrane assembly and energy production 26,33,34. The process of endogenous synthesis is initiated by the transport of mitochondrial citrate into the cytoplasm, where ATP-citrate lyase (ACLY) converts it into acetyl-CoA. Subsequently, acetyl-CoA is carboxylated to form malonyl-CoA by acetyl-CoA carboxylase (ACC), and saturated fatty acids, such as palmitate, are ultimately produced through the condensation-reduction cycle catalyzed by FASN 35,36. This biosynthetic pathway is transcriptionally regulated by sterol regulatory element-binding protein 1 (SREBP1), whose activation is mediated by the phosphoinositide 3- Kinase (PI3K)-protein kinase B (AKT)-mechanistic target of rapamycin complex 1 (mTORC1) signaling pathway 37,38. The newly synthesized saturated fatty acids are then transformed into monounsaturated fatty acids (MUFAs), such as oleate, through the enzymatic activity of stearoyl-CoA desaturase (SCD), thus maintaining the physical properties of membrane lipids 39,40.

The exogenous uptake of fatty acids is contingent upon the proficient operation of transmembrane transport systems. CD36, a fatty acid transporter predominantly localized in the plasma membrane, facilitates the influx of long-chain fatty acids. Under hypoxic conditions, the expression of fatty acid-binding proteins (FABP) is upregulated, thereby enhancing the intracellular trafficking of fatty acids to metabolic compartments 41-43. Exogenously acquired fatty acids require activation to acyl-CoA esters by acyl-CoA synthetase long-chain (ACSL) prior to their entry into downstream metabolic pathways 44-46. These acyl-CoA derivatives are subject to distinct catabolic fates: long-chain acyl-CoA is conveyed into the mitochondrial matrix via CPT1A where it undergoes FAO, yielding acetyl-CoA that subsequently enters the tricarboxylic acid (TCA) cycle for ATP production. This process is transcriptionally upregulated by peroxisome proliferator-activated receptor α (PPARα) under glucose-deficient conditions, thereby establishing a critical alternative energy-generating pathway. Very long-chain acyl-CoA molecules undergo carbon chain shortening within peroxisomes to form medium-chain acyl-CoA, which is subsequently transferred to mitochondria for further oxidation. Additionally, certain fatty acids are hydroxylated at the ω-terminus by endoplasmic reticulum cytochrome P450 enzymes (e.g., CYP4A11) and ultimately oxidized to dicarboxylic acids that enter the FAO pathway 46,47.

This metabolic plasticity permits tumor cells to dynamically adjust fatty acid fluxes in response to their nutritional and energetic demands. During periods of active biosynthesis, fatty acids function as precursors for membrane lipids and are integral to the synthesis of PLs and triacylglycerols. Conversely, under conditions of heightened energy demand, catabolic pathways are swiftly upregulated to facilitate ATP production. Consequently, the fundamental principle of fatty acid metabolic reprogramming is its function as a highly adaptable resource allocation system, providing essential precursors for membranes and stored lipids, which is crucial for tumor survival and proliferation 34,48.

### Triglyceride metabolic reprogramming

Triglycerides (TAGs) serve as the principal storage form of fatty acids, acting as a metabolic buffer through the accumulation of LDs within tumor cells. LDs are subcellular organelles characterized by a neutral lipid core surrounded by a phospholipid monolayer. The surface of these droplets is anchored by perilipin proteins (PLIN1-5), which regulate lipase access to the lipid core via steric hindrance, thus maintaining the equilibrium between lipid storage and lipolysis 49-51. The biogenesis of LDs commences at the endoplasmic reticulum (ER) membrane, where diacylglycerol (DAG) is converted into TAG by the enzymatic activity of diacylglycerol acyltransferases 1/2 (DGAT1/2). The newly formed TAG droplets are enveloped by a phospholipid monolayer and subsequently bud off from the ER to develop into mature LDs 52.

Under conditions of microenvironmental stress, LDs undergo dynamic remodeling. Hypoxia induces the upregulation of the LD-associated protein hypoxia-inducible LDs-associated protein (HILPDA) through the activation of hypoxia-inducible factor-1α (HIF-1α), which subsequently inhibits adipose triglyceride lipase (ATGL) and facilitates the accumulation of LDs. Upon reoxygenation, the phosphorylation of the LD surface protein PLIN2 reduces the steric hindrance of ATGL, thereby initiating rapid TAG hydrolysis 53-56. Lipolysis occurs through a meticulously regulated enzymatic cascade: ATGL catalyzes the hydrolysis of TAG to yield DAG and free fatty acids (FFAs); DAG is further hydrolyzed to monoacylglycerol (MAG) and FFAs by hormone-sensitive lipase (HSL); finally, monoacylglycerol lipase (MGL) cleaves MAG into glycerol and FFAs 1,49,57. The liberated FFAs are swiftly transported into mitochondrial FAO, providing acetyl-CoA for the TCA cycle and thereby supporting ATP production 58. In addition to their role in energy provision, LDs function as crucial metabolic centers for the synthesis of lipid mediators. Phospholipase A2 (PLA2), which is abundant on the LD surface, catalyzes the hydrolysis of membrane PLs to produce lysophosphatidylcholine (LPC). Concurrently, lipoxygenase facilitates the conversion of PUFAs into pro-inflammatory mediators, such as hydroxyeicosatetraenoic acids 59. Furthermore, cholesteryl esters (CEs) stored in the LDs core are synthesized by acyl-CoA cholesterol acyltransferase 1 (ACAT1/SOAT1), with their accumulation being transcriptionally regulated via the SREBP2 pathway 60,61. This complex role in coordinating energy storage, synthesizing signaling precursors, and maintaining cholesterol homeostasis via the storage of cholesteryl esters highlights the adaptability of lipid droplets as organelles, thereby enabling tumor cells to effectively respond to metabolic fluctuations.

### Phospholipid metabolic reprogramming

PLs serve as essential structural constituents of cellular membranes and undergo both compositional remodeling and metabolic reprogramming within tumor cells. Notably, alterations in glycerophospholipid metabolism are predominantly characterized by modifications in the saturation levels of phosphatidylcholine (PC) 32,62-64. The upregulation of lysophosphatidylcholine acyltransferase 1 (LPCAT1) facilitates the preferential incorporation of saturated fatty acids, such as palmitic acid, into the sn-2 position of PC. This process results in a highly saturated membrane lipid environment that significantly affects membrane fluidity and the conformation of transmembrane proteins 65. Simultaneously, the phospholipase D (PLD) family, including PLD2, is frequently overexpressed in various cancer tissues, where it catalyzes the hydrolysis of PC to produce phosphatidic acid (PA). Subsequently, phosphatidic acid phosphatase converts PA into DAG, thereby providing precursors for the recycling of glycerophospholipids. Additionally, both PA and DAG act as signaling lipid precursors, playing a crucial role in maintaining intracellular signaling homeostasis 66-69.

The reprogramming of sphingolipid metabolism is primarily focused on the chain-length-specific regulation of ceramides (Cer) 70-72. Distinct members of the ceramide synthase (CerS) family, such as CerS1, CerS5, and CerS6, demonstrate unique substrate specificities: CerS5 and CerS6 preferentially utilize palmitoyl-CoA to produce C16-Cer, whereas CerS1 predominantly synthesizes C18-Cer. These variations in acyl chain length significantly impact their metabolic destinies 73. Cer can be hydrolyzed by ceramidase to produce sphingosine, which is subsequently phosphorylated by sphingosine kinase 1 (SPHK1) to generate sphingosine-1-phosphate (S1P) 74-77. S1P is exported extracellularly via transporters such as SPNS2 and activates downstream signaling pathways upon binding to G protein-coupled receptors. Intracellularly, S1P can be irreversibly cleaved by sphingosine-1-phosphate lyase (SGPL1) 78,79. The relative rates of synthesis and degradation of both Cer and S1P collectively regulate their dynamic equilibrium, serving as a molecular switch that modulates cellular stress responses.

In cancer, lysophospholipid metabolism undergoes substantial reprogramming. The enzyme PLA2 facilitates the hydrolysis of PC, resulting in the production of LPC, which is subsequently converted into lysophosphatidic acid (LPA) by autotaxin (ATX/ENPP2) 59,80. As an extracellular signaling molecule, LPA engages downstream signaling pathways through its interaction with six identified G protein-coupled receptors (LPAR1-6). Intracellularly, LPA can be reincorporated into PC via acyltransferases such as LPCAT1 and LPCAT2 81-84. This recycling mechanism positions lysophospholipids as a pivotal interface between membrane homeostasis and signal transduction.

### Cholesterol metabolic reprogramming

In tumor cells, cholesterol homeostasis is reprogrammed through both enhanced endogenous synthesis and increased exogenous uptake. The endogenous synthesis is regulated by the transcriptional cascade involving sterol regulatory element-binding protein 2 (SREBP2) 85-87. Initially, the SREBP2 precursor forms a complex with SREBP cleavage-activating protein (SCAP) in the ER 88,89. This complex then translocates to the Golgi apparatus, where SREBP2 undergoes sequential proteolytic cleavage by site-1 protease and site-2 protease, resulting in the release of its transcriptionally active form 90,91. The mature SREBP2 subsequently translocates to the nucleus, where it binds to sterol response elements (SREs) and promotes the expression of rate-limiting enzymes in the mevalonate pathway, such as 3-hydroxy-3-methylglutaryl-CoA reductase (HMGCR). This process facilitates the multi-step conversion of acetyl-CoA to cholesterol 85,92.

The uptake of exogenous cholesterol is enhanced through the upregulation of the low-density lipoprotein receptor (LDLR), which facilitates the endocytosis of circulating low-density lipoprotein (LDL). Subsequent hydrolysis by lysosomal acid lipase releases free cholesterol. Intracellular levels of free cholesterol are stringently regulated by ACAT1/SOAT1, which converts excess free cholesterol into CEs for storage specifically within LDs. The accumulation of CEs serves as an indicator of disrupted cholesterol homeostasis and is transcriptionally regulated by the SREBP2 pathway 93-95.

Cholesterol metabolites exert distinct regulatory functions in tumor cells. Oxysterols, such as 27-hydroxycholesterol, are produced through the cytochrome P450 enzyme CYP27A1-mediated hydroxylation of cholesterol. As endogenous ligands for liver X receptors (LXR), oxysterols upregulate the expression of cholesterol efflux transporters, including ATP-binding cassette transporter A1 (ABCA1), thereby providing negative feedback to suppress SREBP2 activity 96,97. In the bile acid synthesis pathway, cholesterol is hydroxylated by cholesterol 7α-hydroxylase (CYP7A1) to form 7α-hydroxycholesterol, which is subsequently metabolized into bile acids. This pathway is frequently downregulated in hepatocellular carcinoma, resulting in impaired conversion of cholesterol to bile acids 98,99.

The subcellular distribution of cholesterol undergoes significant metabolic reprogramming. Enrichment of cholesterol in plasma membrane lipid rafts modulates the efficiency of transmembrane signaling by affecting the conformation and clustering of receptors, including EGFR and transforming growth factor-beta (TGF-β) receptors. Simultaneously, increased cholesterol levels in mitochondrial membranes modify membrane permeability, thereby contributing to apoptosis resistance 100-104. This comprehensive metabolic remodeling positions cholesterol as a pivotal element in regulating both the structural integrity and functional dynamics of tumor cell membranes.

In a critical examination, it is evident that the reprogrammed lipid modules, encompassing fatty acid, triglyceride, phospholipid, and cholesterol metabolism, do not function independently but rather as a cohesive and integrated network 1. Fatty acids are essential precursors not only for energy production but also for the synthesis of phospholipid membranes and the storage of triglycerides. LDs, which serve as storage sites for triglycerides, also play a pivotal role by sequestering cholesterol esters, thus modulating cholesterol levels and establishing a direct link between triglyceride storage and cholesterol homeostasis. The remodeling of phospholipids, significantly influenced by the saturation state of fatty acids and the enrichment of cholesterol within specific membrane domains such as lipid rafts, actively shapes the physical and signaling characteristics of cellular membranes 105. This interconnected system enables tumor cells to efficiently allocate lipid resources, synchronize energy storage with membrane synthesis and signaling, and adapt to the variable demands of their microenvironment. Functionally, fatty acids act as a central metabolic hub that integrates energy production, membrane biosynthesis, and lipid storage, thereby linking triglyceride, phospholipid, and cholesterol metabolism into a coordinated adaptive network.

## Reprogramming of Lipid Metabolism Facilitates the Malignant Phenotype in Tumors

**Figure 3:**
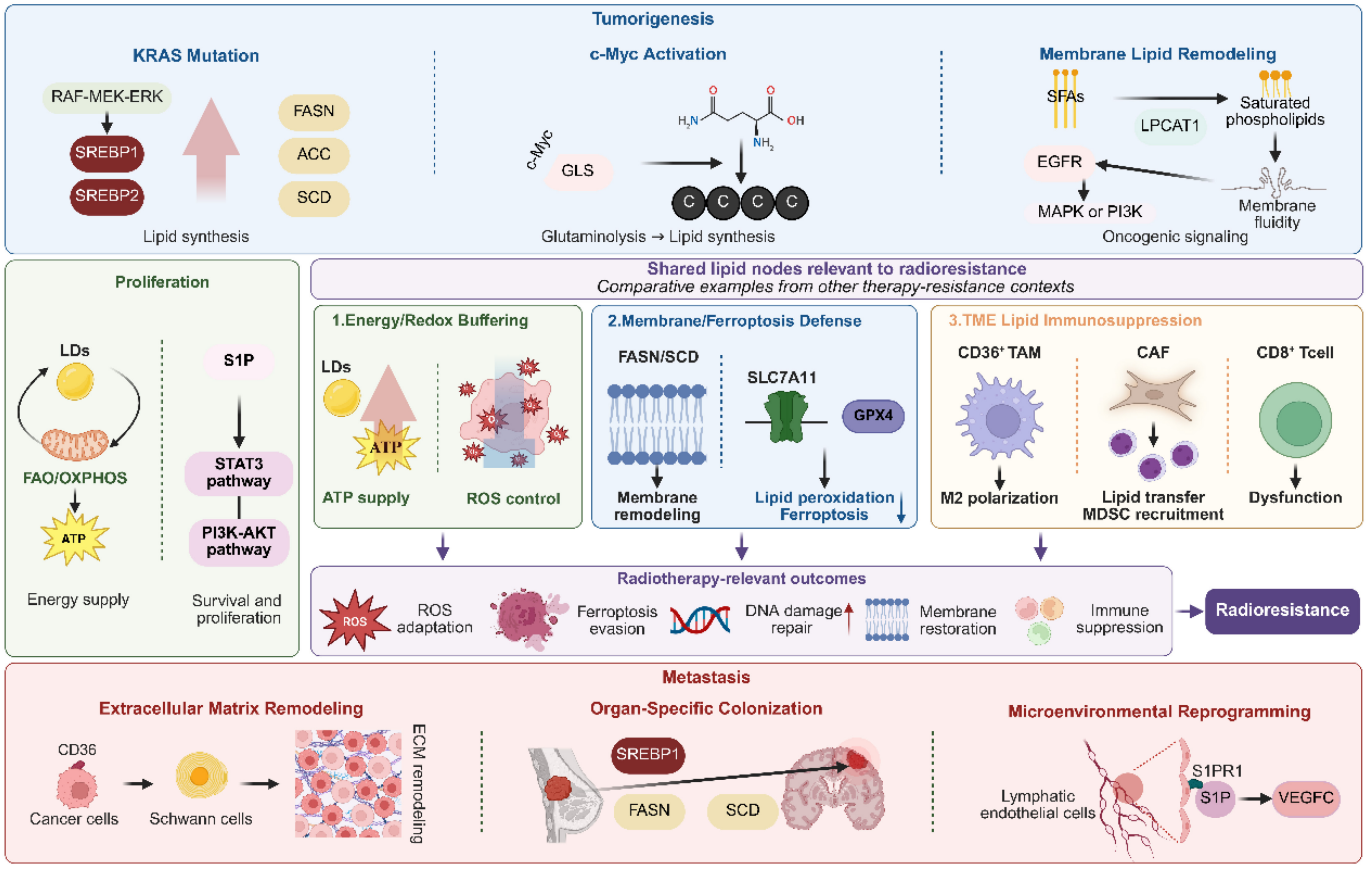
**Lipid Metabolic Rewiring Propels Malignant Pathogenesis.** Lipid metabolic reprogramming promotes malignant phenotypes and generates shared lipid nodes relevant to radiotherapy resistance. Oncogene-driven lipogenesis, c-Myc-dependent glutaminolysis, membrane lipid remodeling, LD/FAO/OXPHOS activity, and S1P signaling support tumorigenesis and proliferation. Resistance mechanisms from chemotherapy, targeted therapy, and immunotherapy are presented as comparative examples that converge on radiotherapy-relevant processes, including redox buffering, ferroptosis evasion, membrane restoration, DNA damage repair, and lipid-mediated TME immunosuppression. Lipid reprogramming also promotes metastasis through CD36-dependent ECM remodeling, organ-specific lipid adaptation, and S1P/S1PR1-mediated microenvironmental remodeling. Created with BioRender.com.

Lipid metabolic reprogramming contributes to malignant phenotypes, but its relevance to radiotherapy depends on how these programs alter radiation-induced damage and recovery. Oncogenic control of lipid synthesis, uptake, storage, and oxidation can influence membrane composition, redox buffering, ferroptosis susceptibility, DNA damage repair, and survival signaling after IR. These same lipid states also emerge during tumor initiation, proliferation, invasion, and metastasis, thereby linking malignant progression to subsequent differences in radiosensitivity and radioresistance. **Figure 3** illustrates how lipid programs that support tumorigenesis, proliferation, and metastasis may also converge on radiotherapy-relevant resistance nodes.

### The initiation of lipid synthesis represents an early metabolic hallmark of tumorigenesis

In the early stages of tumorigenesis, the activation of oncogenes and the inactivation of tumor suppressor genes lead to a direct reprogramming of lipid metabolism. Mutations in the oncogene KRAS activate the RAF-MEK-ERK signaling pathway, which promotes the proteolytic maturation and nuclear translocation of SREBPs. This process transcriptionally upregulates enzymes such as FASN, ACC, and SCD 106-109. Similarly, the activation of the PI3K-AKT pathway, resulting from PIK3CA mutations or the loss of PTEN, leads to the phosphorylation and inhibition of glycogen synthase kinase 3 beta (GSK3β). This inhibition alleviates the suppression of SCAP, thereby enhancing the processing of SREBP1/2 110-113. This SREBP-mediated lipogenic program is evident in precancerous lesions; for example, KRAS mutations cause early overexpression of ATP citrate lyase (ACLY) and FASN in pancreatic intraepithelial neoplasia (PanIN), providing essential membrane components necessary for malignant transformation 107,114-116.

The amplification or overexpression of the c-Myc oncogene significantly increases lipogenic demand. c-Myc directly interacts with the promoter of glutaminase (GLS), thereby mitigating transcriptional repression by miR-23a/b and promoting glutaminolysis to provide carbon precursors essential for fatty acid synthesis 117-120. Simultaneously, c-Myc enhances the expression of glutamine synthetase (GLUL), facilitating autonomous nitrogen production under conditions of glutamine deficiency and supporting the synthesis of nucleotides and hexosamines 117,121,122. Importantly, the inactivation of p53 enhances the flux through the pentose phosphate pathway by alleviating the allosteric inhibition of glucose-6-phosphate dehydrogenase (G6PD), thus supplying the NADPH necessary for fatty acid synthesis 123-125. This orchestrated activation of multiple lipid synthesis pathways establishes an early "lipid addiction" phenotype in nascent tumors.

The reconfiguration of membrane lipid composition plays a crucial role in the amplification of oncogenic signaling. In KRAS-mutant cells, LPCAT1 is overexpressed, facilitating the incorporation of saturated fatty acids, such as palmitate, into the sn-2 position of PC, thereby generating highly saturated PLs 65,126-128. This increase in lipid saturation within the membrane decreases fluidity, promotes the clustering and activation of receptor tyrosine kinases, including EGFR, and enhances downstream MAPK and PI3K signaling pathways 129. Simultaneously, the upregulation of cholesterol synthesis enriches lipid raft microdomains, which serve as platforms for oncogenic signaling by receptors such as HER2 and c-Met, thereby facilitating cell cycle progression 130-132. Under conditions of genotoxic stress, the synthesis of MUFAs, such as oleate, confers protection to precancerous cells against apoptosis by mitigating ER stress and lipotoxicity 133,134. Consequently, lipid metabolic reprogramming is not merely a secondary feature of tumorigenesis, but rather an active contributor to malignant transformation.

### Lipid metabolic reprogramming serves as a pivotal mechanism in the proliferation of tumors

During the proliferative phase, tumor cells exhibit an increased demand for lipids that extends beyond mere membrane construction to include bioenergetic supply and the maintenance of signaling networks. The persistent activation of the PI3K-AKT pathway orchestrates lipid synthesis and energy metabolism. Specifically, AKT phosphorylates ACLY at the Ser455 residue, mitigating the inhibitory phosphorylation mediated by GSK3β and thereby promoting the production of cytoplasmic acetyl-CoA. This acetyl-CoA acts as a precursor for the synthesis of fatty acids and cholesterol 135,136. Simultaneously, AKT activates NAD kinase 1 (NADK1), facilitating the conversion of NAD⁺ to NADP⁺ and augmenting the NADPH pool, which is crucial for supporting reductive biosynthetic processes such as fatty acid elongation 135,137. This AKT-mediated metabolic reprogramming allows for sustained cellular proliferation even in conditions of limited growth factor availability.

LDs serve as central sites for the dynamic storage and release of lipids, demonstrating significant adaptability during cellular proliferation. Under hypoxic conditions, there is an enhanced accumulation of TAG within LDs 36,138, while reoxygenation promotes TAG hydrolysis, providing acetyl-CoA for the TCA cycle 31. In glucose-restricted environments, such as pre-metastatic lung niches, FAO emerges as the predominant pathway for ATP production, thereby facilitating cytoskeletal reorganization and enhancing migratory capabilities 139,140. CEs stored in LDs play a crucial role in maintaining free cholesterol homeostasis through ACAT1/SOAT1-mediated esterification and hydrolysis processes, effectively bypassing SREBP2 negative feedback and supporting membrane biogenesis 141,142.

The reprogramming of sphingolipid metabolism further facilitates cellular proliferation by modulating the balance between pro-survival and pro-apoptotic signals. SPHK1 is frequently overexpressed in tumors characterized by PI3K-AKT pathway activation, where it catalyzes the conversion of sphingosine to S1P. S1P, through autocrine and paracrine interactions with G protein-coupled receptors (S1PR1-5), activates the PI3K-AKT and STAT3 signaling pathways, inhibits Bax activation, and upregulates the expression of the anti-apoptotic protein Mcl-1 143-145. Furthermore, the oncogene c-Myc contributes to the reduction of pro-apoptotic Cer levels by downregulating the expression of the CerS family, thereby diminishing apoptotic constraints 146,147. In tumors associated with obesity, fatty acids released from adipose tissue lead to an upregulation of SPHK1 in tumor cells, which in turn increases S1P production, stimulates proliferation, and recruits tumor-promoting macrophages 148,149. This resultant imbalance between Cer and S1P underscores the role of sphingolipid metabolism as a critical regulatory mechanism that enables tumor cells to circumvent growth suppression.

Mitochondrial oxidative phosphorylation (OXPHOS) and FAO play a pivotal role in providing the necessary energy for rapid cellular proliferation. In estrogen receptor-positive breast cancer, estrogen enhances mitochondrial biogenesis by upregulating factors such as PGC-1α and TFAM, as well as OXPHOS complex subunits like ND1 and COX-IV, thereby augmenting the oxidative metabolism of fatty acid-derived acetyl-CoA 150-152. Triple-negative breast cancer (TNBC) demonstrates a pronounced reliance on OXPHOS; under the stress of chemotherapy, the inhibition of CPT1A-mediated fatty acid transport can act synergistically with chemotherapeutic agents to enhance treatment efficacy 153,154. Similarly, glioma stem cells sustain their self-renewal and drug resistance capabilities by maintaining elevated mitochondrial activity and OXPHOS protein expression 155-157. Consequently, the integration of lipid and energy metabolism establishes a comprehensive network that supports tumor proliferation.

### Reprogramming of lipid metabolism facilitates tumor invasion and metastasis

During metastasis, tumor cells undergo reprogramming of lipid uptake, synthesis, and modification to facilitate the colonization of distant organs. The long-chain fatty acid transporter CD36 is notably overexpressed in metastasis-initiating cells. In the context of oral squamous cell carcinoma, CD36-positive cells exhibit stem-like characteristics; upon the uptake of palmitic acid from the TME, these cells activate a pro-metastatic transcriptional program and promote extracellular matrix remodeling by inducing phenotypic changes in intratumoral Schwann cells 158-160. In obese patients, elevated serum palmitic acid levels enhance melanoma resistance to targeted therapy via CD36, thereby accelerating metastatic recurrence 158,161,162. Importantly, lymphatic and hematogenous metastasis occur within distinct lipid environments: lymph fluid is rich in oleic acid (18:1), which protects circulating melanoma cells from ferroptosis by inhibiting PUFA-mediated lipid peroxidation, thus enhancing lymph node metastatic efficiency 158,163.

Organ-specific colonization is contingent upon the precise adaptation of lipid metabolic pathways. Brain metastasis presents distinct metabolic challenges due to the lipid-deficient nature of the interstitial fluid, compelling tumor cells to depend significantly on *de novo* lipid synthesis. In the context of brain metastases originating from breast cancer, there is a sustained overexpression of FASN and SCD through continuous activation of SREBP1. This process facilitates the synthesis of palmitate and its subsequent desaturation to oleate, thereby preventing ER stress and preserving membrane fluidity 164-166. In HER2-positive breast cancer brain metastases, the FABP7 is upregulated, enhancing the uptake of ω-3 PUFAs, such as docosahexaenoic acid (DHA), from the microenvironment and activating peroxisome proliferator-activated receptor gamma (PPARγ) signaling to support the formation of the metastatic niche 167,168. In contrast, liver metastasis employs a different strategy: within the lipid-abundant hepatic environment, colorectal cancer cells downregulate FASN and upregulate CD36 to facilitate the uptake of fatty acids released by hepatic stellate cells, thereby fueling energy production through CPT1A-mediated FAO 169. Breast cancer cells metastasizing to the lungs adapt to glucose scarcity by utilizing FAO, generating acetyl-CoA via CPT1A to acetylate the nuclear factor kappa-light-chain-enhancer of activated B cells (NF-κB) p65 subunit at lysine 310 and drive expression of pro-metastatic genes 170.

### Shared lipid nodes relevant to radiotherapy resistance

Lipid-dependent resistance becomes particularly relevant in radiotherapy because ionizing radiation imposes simultaneous oxidative, membrane, metabolic, and immune stresses on tumor cells and the TME. Under these conditions, resistance-associated lipid programs may influence IR-induced ROS injury, lipid peroxidation and ferroptosis, DNA damage repair, membrane restoration, and anti-tumor immunity. Evidence from chemotherapy, targeted therapy, and immunotherapy settings is therefore used to identify shared lipid nodes, including FAO-mediated energy support, redox buffering, membrane remodeling, ferroptosis suppression, and lipid-driven immunosuppression within the TME 171-180.

Resistance to immunotherapy is closely linked to lipid-mediated immunosuppression within the TME. TAMs expressing CD36 internalize lipids from tumor-derived exosomes, subsequently adopting a pro-metastatic M2-like phenotype and inhibiting T cell function through the secretion of immunosuppressive factors such as TGF-β 179. In the context of hepatocellular carcinoma, CD36-positive CAFs attract CD33-positive myeloid-derived suppressor cells (MDSCs), thereby creating an immune-privileged niche 158. Additionally, farnesyl pyrophosphate (FPP), synthesized by prostate cancer cells via the mevalonate pathway, facilitates the prenylation of Ras proteins, which in turn sustains pro-survival signaling and hinders T cell infiltration 180.

IR significantly alters the lipid network within tumors by causing direct DNA damage and generating ROS indirectly. Preclinical research suggests that radiation activates the SREBP pathway, which enhances cholesterol synthesis for membrane repair and upregulates fatty acid desaturases (FADSs), such as SCD, to increase the MUFA content in membranes. This adaptation serves to mitigate radiation-induced lipid peroxidation. Such metabolic adaptations may undermine the effectiveness of radiotherapy and provide a theoretical basis for developing novel radiosensitization strategies that target lipid metabolism.

## Mechanisms of Cell Death Induced by Ionizing Radiation: Disruption of Lipid Homeostasis in Tumors

**Figure 4:**
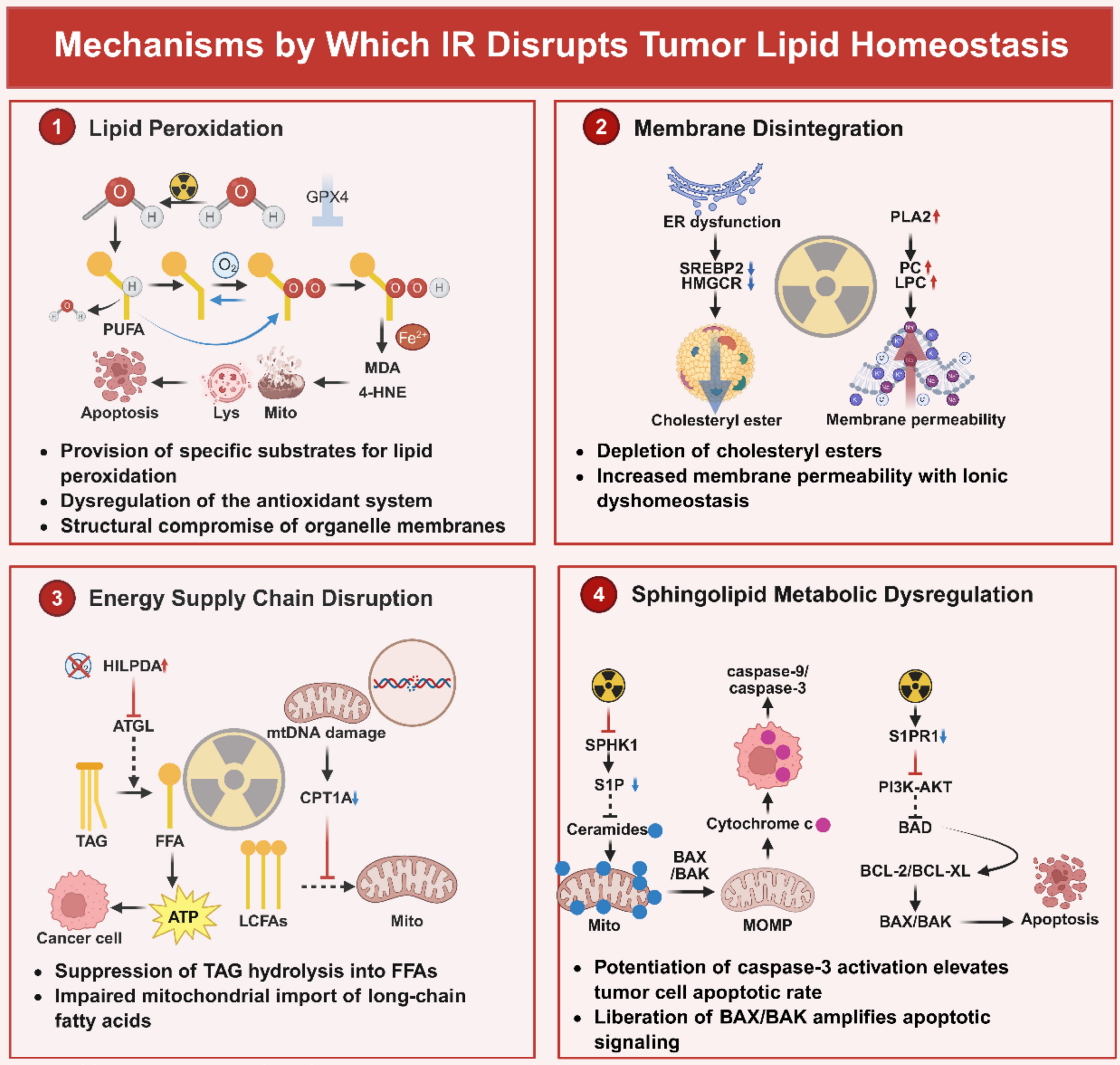
** IR Disrupts Tumor Viability Through Strategic Lipid Homeostasis Subversion.** This graphical abstract illustrates the mechanisms by which IR facilitates tumor eradication through the disruption of lipid homeostasis across four synergistic pathways. Firstly, lipid peroxidation is executed by radiolytic ·OH targeting phospholipid-bound polyunsaturated fatty acids, specifically AA and AdA esterified by ACSL4. This process leads to chain-amplified peroxidation, resulting in the accumulation of cytotoxic 4-HNE and MDA, thereby enhancing ferroptosis. Secondly, membrane structural collapse is initiated by IR-induced impairment of SREBP2 processing, which suppresses HMGCR-dependent cholesterol biosynthesis and leads to the disintegration of lipid rafts. Concurrently, PLA2-mediated hydrolysis of phosphatidylcholine to LPC increases bilayer permeability. Additionally, downregulation of LPCAT1 elevates the proportion of PUFAs in the membrane, increasing fluidity and destabilizing receptor tyrosine kinase clustering and signal transduction. The disruption of energy supply is facilitated by the overexpression of HILPDA induced by radiation, which inhibits ATGL-mediated TAG hydrolysis in LDs. This inhibition prevents the mobilization of FFAs necessary for FAO during glucose deprivation. Concurrently, damage to mtDNA suppresses the expression of CPT1A, impairing the import of fatty acids into mitochondria. Additionally, the oxidation of the ACO2 cluster halts the flux of the TCA cycle, leading to an ATP crisis. Within 24 hours post-IR, sphingolipid-driven apoptotic priming is characterized by the inhibition of SPHK1, resulting in the depletion of the pro-survival molecule S1P and an increase in mitochondrial ceramide. This elevation directly facilitates the oligomerization of BAX/BAK, inducing MOMP and the release of cytochrome C, which activates the caspase-9/3 cascades. Simultaneously, the internalization of S1PR1 disrupts PI3K-AKT survival signaling, thereby relieving BAD-mediated inhibition of BCL-2/BCL-XL. Collectively, these IR-induced disruptions in lipid networks, encompassing peroxidative membrane rupture, loss of structural integrity, bioenergetic failure, and engagement of apoptotic pathways, constitute a multimodal lethality paradigm that can be exploited for radiosensitization. Created with BioRender.com.

Radiation-induced lipid disruption follows a temporally ordered process. Initial ionizing radiation rapidly generates ROS through water radiolysis, causing immediate oxidative damage to PUFA-containing membrane lipids and initiating lipid peroxidation 181,182. This occurs prior to transcriptional changes. Subsequently, stress signaling pathways such as ATM are activated, leading to alterations in antioxidant and lipid metabolic gene expression 183-185. Mitochondrial damage is further amplified as a downstream consequence of sustained oxidative stress and may contribute to the propagation of lipid peroxidation and ferroptotic signaling 185-187. Progressive lipid peroxidation, together with glutathione depletion and GPX4 inactivation, ultimately drives membrane damage and ferroptotic cell death. Notably, cellular susceptibility to this process is largely determined by baseline lipid composition, particularly PUFA enrichment regulated by ACSL4 188-190. The mechanisms by which lipid homeostasis is disrupted are illustrated in **Figure 4**.

### Lipid peroxidation directly triggers radiotherapy-induced ferroptosis

Ferroptosis represents a regulated form of cell death that is driven by iron-dependent lipid peroxidation and is characterized by the disruption of mitochondrial cristae, the inactivation of glutathione peroxidase 4 (GPX4), and the accumulation of lipid peroxides such as 4-hydroxynonenal (4-HNE) 191-195. The lipid peroxidation cascade triggered by ionizing radiation constitutes a DNA damage-independent cytotoxic pathway that complements traditional radiation-induced DNA damage responses. This pathway demonstrates selective cytotoxicity against therapy-resistant malignancies, including those harboring KRAS mutations 196,197.

The radiation-induced disruption of lipid homeostasis is initiated by the ROS-mediated peroxidation of PUFAs. Hydroxyl radicals, generated through water radiolysis during IR exposure, abstract hydrogen atoms from long-chain PUFAs, such as arachidonic acid (AA; C20:4) and adrenic acid (AdA; C22:4), within membrane PLs, resulting in the formation of lipid radicals (L·). These radicals rapidly react with molecular oxygen to form lipid peroxyl radicals (LOO·), thereby initiating a chain-propagating peroxidation reaction 182. At the molecular level, this process involves the oxidative cleavage of PUFA chains embedded within the phospholipid bilayer. Ether PLs, particularly phosphatidyl ethanolamines (PEs), are highly susceptible to oxidative damage due to their high content of PUFAs 188,198. The enzyme Acyl-CoA Synthetase Long-chain family member 4 (ACSL4) plays a pivotal catalytic role by esterifying free AA and AdA into phosphatidylethanolamine-conjugated PUFAs (PE-PUFAs), thereby providing specific substrates for lipid peroxidation 189,190. Preclinical studies have shown that the knockout of ACSL4 significantly reduces the incorporation of PUFAs into cellular membranes and imparts resistance to radiation-induced ferroptosis 189,199. Furthermore, elevated ACSL4 expression is positively associated with enhanced survival rates in patients with irradiated esophageal cancer 200,201.

Radiation-induced lipid peroxidation is exacerbated by the disruption of antioxidant defenses. IR activates the ataxia-telangiectasia mutated (ATM) gene, which downregulates the expression of solute carrier family 7 member 11 (SLC7A11), thereby impairing the cystine uptake mediated by system Xc⁻ 183-185. The resultant cystine deficiency constrains GSH synthesis, leading to a depletion of intracellular GSH reserves 184,202. Given that GSH serves as a critical cofactor for GPX4, its depletion results in the inactivation of GPX4, the key enzyme in mammalian cells capable of reducing membrane phospholipid hydroperoxides (PL-OOH). This inactivation leads to the irreversible accumulation of toxic lipid peroxides, such as malondialdehyde (MDA) and 4-HNE 203,204. Notably, the GPX4 inhibitor RSL-3 simulates radiation-induced ferroptosis by directly binding to the selenocysteine residue within GPX4's catalytic site 205,206. Furthermore, radiotherapy induces the secretion of interferon-γ (IFNγ) by CD8⁺ T cells, which activates STAT1 signaling and further suppresses SLC7A11, thereby creating an immunometabolic positive feedback loop that intensifies ferroptosis 207.

Accumulated lipid peroxides cause significant damage to organelle membranes. Transmission electron microscopy demonstrates notable mitochondrial condensation in irradiated tumor cells, which is distinct from the apoptotic morphology induced by chemotherapy 186,187. The aldehyde 4-HNE, a product of lipid peroxidation, covalently modifies sulfhydryl groups on membrane proteins, leading to disrupted ion channel function, increased calcium influx, and lysosomal membrane permeabilization 208. In addition to its direct cytotoxic effects, 4-HNE serves as a potent signaling molecule that mediates downstream responses to radiation-induced damage. It is capable of forming covalent adducts with key signaling proteins, thereby modulating critical pathways such as the NRF2 antioxidant response, NF-κB-mediated inflammation and survival signaling, and the PI3K/AKT growth pathways. This signaling activity can significantly influence cellular fate decisions following irradiation, potentially contributing to adaptive survival mechanisms or exacerbating genotoxic stress, contingent upon the context and extent of 4-HNE generation 209,210. Ultimately, prolonged oxidative stress compromises plasma membrane integrity, culminating in necrotic cell death.

Clinical evidence highlights the critical influence of lipid peroxidation on the efficacy of radiotherapy. Notably, neoadjuvant radiotherapy results in a marked increase in 4-HNE immunopositivity within esophageal tumor tissues, which is associated with enhanced pathological response and improved overall survival 3,211. Mechanistic investigations utilizing lung cancer patient-derived xenograft (PDX) models reveal that the combination of radiotherapy with ferroptosis inducers, such as SLC7A11 inhibitor, produces synergistic antitumor effects. In contrast, monotherapy approaches demonstrate limited effectiveness, underscoring the specificity of this radiosensitization strategy 212,213. Crucially, the sensitivity to radiation-induced ferroptosis is influenced by the cellular lipid composition: clear cell renal cell carcinoma, characterized by an abundance of PUFAs due to pseudohypoxia-driven upregulation of HILPDA, shows increased sensitivity 214. In contrast, KRAS-mutant tumors activate the NRF2-KEAP1 pathway to upregulate SLC7A11 expression, thereby conferring radioprotection 215,216. These findings lay the molecular groundwork for the personalization of radiotherapeutic strategies based on lipid metabolic phenotypes.

### Disruption of cholesterol-phospholipid homeostasis

IR undermines the structural integrity of tumor cell membranes by disrupting cholesterol biosynthesis and phospholipid remodeling pathways. Radiotherapy impairs ER function and inhibits the proteolytic activation of SREBP2, leading to the downregulation of its downstream target gene, HMGCR 217-219. As the rate-limiting enzyme in cholesterol synthesis, the inhibition of HMGCR disrupts the mevalonate pathway and significantly reduces membrane cholesterol content 220. The depletion of cholesterol directly compromises the structural integrity of lipid raft microdomains, resulting in the disaggregation and impaired endocytosis of receptor tyrosine kinases, such as EGFR and HER2, which are localized within these rafts. This process suppresses their aberrant pro-survival signaling following irradiation 221. Preclinical studies indicate that the combination of the HMGCR inhibitor pitavastatin with radiotherapy enhances radiosensitivity in melanoma models by promoting DNA double-strand breaks and inducing cellular senescence 222.

These seemingly divergent effects should be interpreted within a context-dependent framework, as acute radiation injury, inter-fraction adaptation, and chronically radioresistant states may exhibit distinct patterns of cholesterol remodeling. Concurrently, radiation exposure leads to the activation of PLA2, which catalyzes the hydrolysis of membrane PC to produce LPC 223. As an amphiphilic lipid mediator, LPC integrates into the lipid bilayer, thereby enhancing passive membrane permeability and disrupting ion homeostasis 223,224. In ovarian cancer cells, the accumulation of LPC contributes to therapy resistance by inhibiting the Fas/FasL apoptotic pathway 225,226. Additionally, radiotherapy results in the downregulation of LPCAT1, an enzyme responsible for the incorporation of saturated fatty acids into PC 227. Under normal physiological conditions, LPCAT1 plays a critical role in maintaining the homeostatic balance of saturated and unsaturated fatty acids (SFA/UFA) within PLs; its suppression leads to an increased relative abundance of PUFAs in cellular membranes, causing an abnormal rise in membrane fluidity and increased vulnerability to oxidative damage 127,227. This dual dysregulation, characterized by cholesterol depletion and phospholipid imbalance, compromises the membrane stability advantage that tumors acquire through pre-radiotherapy cholesterol and phospholipid remodeling, thereby depriving tumor cells of adaptive resistance to radiation stress 228.

### Depletion of lipid droplets and inhibition of FAO

Radiotherapy disrupts the lipid-dependent energy metabolism of tumors by depleting LDs storage pools and impairing mitochondrial FAO. The radiation-induced hypoxic TME upregulates HILPDA 55,229, which directly inhibits the activity of ATGL 56,230, thereby suppressing the hydrolysis of TAGs within LDs into FFAs 231,232. Under conditions of glucose deprivation, this impairment hinders tumor cells from mobilizing LDs reserves for emergency energy production, resulting in a metabolic crisis 228. In glioblastoma models, radiation enhances tumor survival by inducing FASN-mediated *de novo* lipogenesis and LDs accumulation 233,234. In contrast, in breast cancer, the pharmacological inhibition of DGAT2, a key enzyme in LD biogenesis, using agents such as PF-06424439, increases radiosensitivity by reducing LD content 8,235. Consequently, radiation has the potential to acutely interfere with LD-dependent energy mobilization. In contrast, tumor cells that survive or exhibit radioresistance may subsequently restore or augment LD accumulation as an adaptive response.

Mitochondrial FAO impairment constitutes a significant mechanism by which radiation disrupts cellular energy supply. Radiation-induced damage to mitochondrial DNA (mtDNA) results in the downregulation of CPT1A expression 236. As CPT1A serves as the rate-limiting enzyme in the mitochondrial import of fatty acids, its suppression hinders the transport of long-chain fatty acids into the mitochondria 237,238. In cases of radiotherapy-resistant nasopharyngeal carcinoma, increased expression of CPT1A is significantly associated with poor patient prognosis 13. Conversely, the genetic ablation of CPT1A or CPT2 using CRISPR-Cas9 technology, or the pharmacological inhibition with etomoxir, enhances radiosensitivity in breast cancer by obstructing FAO 239,240. Additionally, radiation impairs the activity of crucial TCA cycle enzymes, such as citrate synthase and aconitase 2 (ACO2). The [4Fe-4S] cluster within the active site of ACO2 is particularly vulnerable to oxidative inactivation induced by radiation; the loss of ACO2 function results in citrate accumulation, impaired isocitrate formation, and ultimately, TCA cycle arrest 241,242. Collectively, these defects cause a collapse in mitochondrial ATP synthesis, resulting in cell death due to energy exhaustion.

Clinical evidence underscores the translational relevance of these mechanisms. Preclinical and mechanistic evidence suggests that targeting the PDK1-mediated glycolytic shift may restore oxidative metabolism, enhance ROS production, and potentially improve radiosensitivity in esophageal squamous cell carcinoma models 243,244.

### Sphingolipid metabolic imbalance promotes apoptosis

IR perturbs the homeostatic equilibrium between Cer and S1P by altering sphingolipid metabolism, thereby enhancing mitochondrial apoptotic signaling. Radiotherapy markedly suppresses SPHK1 activity, leading to a reduction in the synthesis of the pro-survival mediator S1P and mitigating its physiological antagonism toward Cer 245-247. As a pivotal apoptotic messenger, Cer accumulates in the outer mitochondrial membrane within 24 hours following irradiation 247,248, directly facilitating BAX/BAK oligomerization and inducing mitochondrial outer membrane permeabilization (MOMP) 249. This process subsequently triggers the release of cytochrome C into the cytoplasm, thereby initiating the caspase-9/caspase-3 apoptotic cascade 250,251. In glioblastoma models, the combination of exogenous Cer analogs (e.g., C6-Cer) with radiotherapy significantly enhances tumor cell apoptosis through increased activation of caspase-3 252-254.

Simultaneously, radiation leads to the downregulation of the S1P receptor S1PR1 and its related pro-survival signaling pathways 148,255,256. The internalization and subsequent functional loss of this G protein-coupled receptor result in the suppression of downstream PI3K-AKT signaling 145,257,258. The ensuing inactivation of AKT eliminates the phosphorylative inhibition of the pro-apoptotic protein BAD, allowing BAD to bind to BCL-2/BCL-XL and consequently freeing BAX/BAK to enhance the apoptotic signal. Preclinical studies indicate that the overexpression of SPHK1 imparts radioresistance in breast cancer by maintaining the activity of the S1P-S1PR1-AKT axis 259. In contrast, SPHK1 inhibitors, such as SKI-II, alter the Cer/S1P balance in favor of apoptosis by inhibiting S1P production, thereby increasing the sensitivity of colorectal cancer to radiotherapy 259.

IR exerts its antitumor effects by synergistically disrupting lipid peroxidation homeostasis, membrane cholesterol-phospholipid integrity, LDs-mediated energy supply, and sphingolipid-regulated apoptotic pathways. However, it is crucial to acknowledge that tumors can acquire radioresistance through lipid metabolic adaptations. These adaptations include the overexpression of GPX4 to inhibit ferroptosis, upregulation of CPT1A to sustain FAO, and activation of the SPHK1-S1P axis to promote survival signaling 228. This phenomenon will be examined in detail in the subsequent section to identify key molecular targets for future research.

## Mechanisms Underlying Tumor Radioresistance Facilitated by Lipid Metabolic Reprogramming

IR serves as a fundamental antitumor mechanism by disrupting lipid metabolic homeostasis within tumor cells. The aforementioned processes highlight the crucial role of the lipid metabolic network in modulating the response to radiation and suggest potential targets for enhancing radiosensitivity. Nevertheless, tumors, characterized by their high adaptability, exploit inherent metabolic heterogeneity and exceptional plasticity to develop resistance to radiotherapeutic stress. As previously noted, even under direct radiotherapeutic challenge, tumor cells can develop resistance through various lipid metabolic pathways. This resistance is not random but represents an evolutionarily refined survival strategy facilitated by lipid metabolic reprogramming.

Lipid metabolism-driven radioresistance can be understood through several interconnected layers. Some resistant phenotypes arise from pre-existing metabolic features of tumor cells, such as constitutively elevated FASN- or CPT1A-dependent pathways maintained by oncogenic signaling before irradiation 260,261. Others emerge during or after radiotherapy as adaptive responses, including upregulation of CPT1A through the PGC1α/CEBPB axis or activation of the FASN/PGC1α pathway to support membrane repair, redox balance, and energy production under radiation-induced stress 15,105. The biological impact of these pathways varies across tumor lineage, genetic background, oxygenation status, lipid availability, immune composition, radiation dose and fractionation, and previous systemic treatment. In parallel, the TME, including TAMs, Tregs, CAFs, adipocytes, and exhausted T cells, can amplify metabolic adaptation by providing lipids, competing for nutrients, or reshaping immune responses 262,263. Ferroptosis evasion further integrates antioxidant defense, lipid remodeling, iron handling, and metabolic plasticity into a common survival phenotype. These processes interact dynamically, forming a multilayered lipid metabolic network that supports radioresistance.

**Figure 5:**
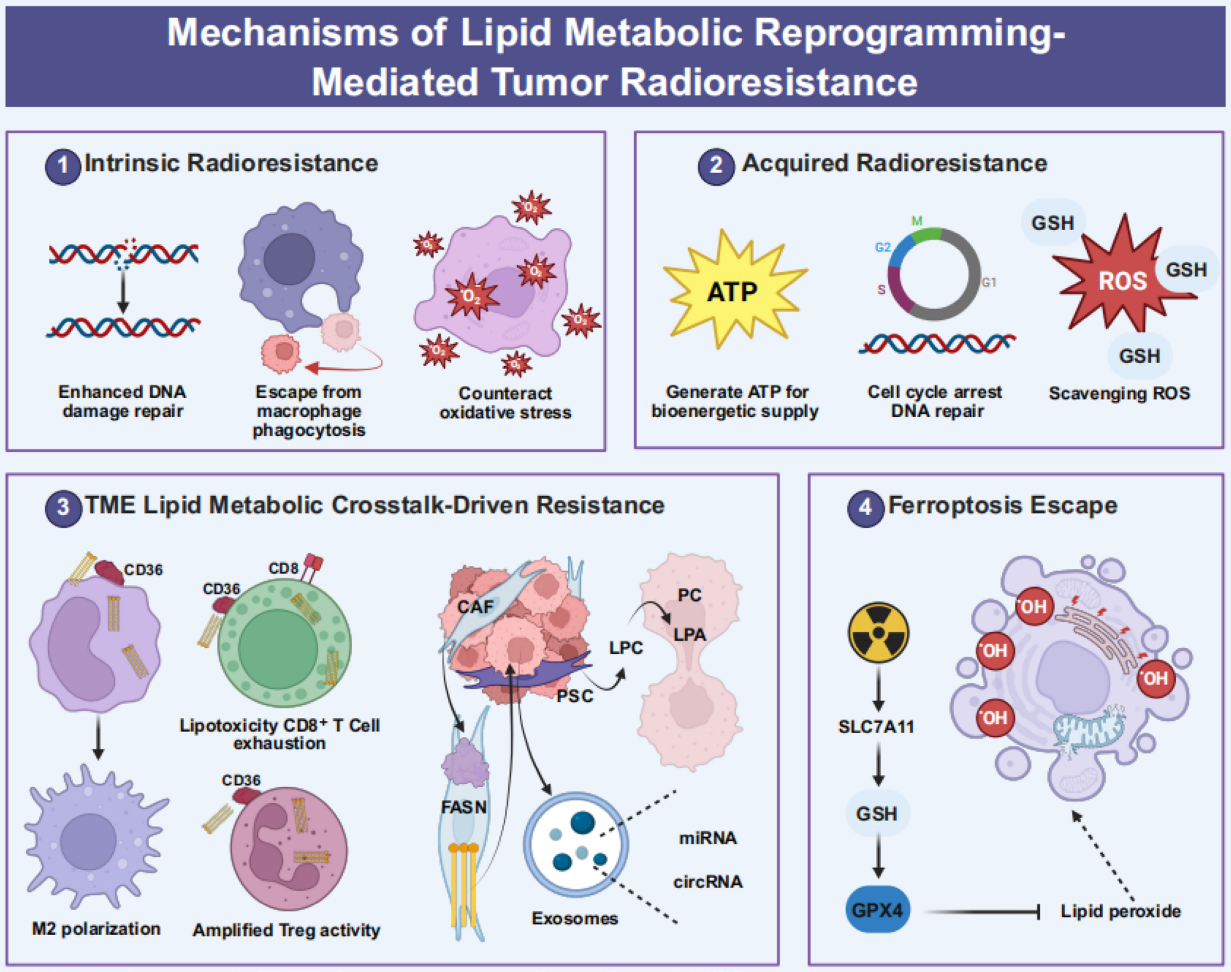
** Lipid Metabolic Enhancement Facilitating Radioresistance.** This graphical abstract illustrates the mechanisms by which lipid metabolism contributes to radioresistance through four interconnected defense strategies. First, intrinsic radioprotection is achieved as ACLY-generated cytosolic acetyl-CoA enhances NHEJ via Ku70 acetylation and supports HR through the stabilization of BRCA1. This is further supported by CPT1A-driven FAO, which maintains ATP production essential for DNA repair processes, and the regulation of FAO metabolites by CD47, which facilitates evasion from phagocytosis. Second, oxidative stress is counteracted through acquired metabolic adaptation, characterized by the upregulation of CPT1A post-irradiation via the PGC1α/CEBPB axis, which accelerates the transfer of fatty acids from lipid droplets to mitochondria, thereby fueling FAO bioenergetics. Concurrently, FASN/PGC1α-induced *de novo* lipogenesis elevates androgen receptor levels, inducing G2/M cell cycle arrest to facilitate DNA damage repair. Additionally, the overproduction of phosphatidylcholine and phosphatidylethanolamine, in synergy with increased GSH levels, effectively scavenges radiation-induced ROS. The immunosuppressive mechanisms driven by the TME involve cancer cell-derived FFAs that exploit CD36-positive TAMs to trigger FAO, STAT6 activation, and Arg1-dependent M2 polarization. Concurrently, CD36-mediated lipid accumulation in CD8⁺ T cells elevates the expression of exhaustion markers PD-1, TIM-3, and LAG-3. CAFs also secrete FFAs in a FASN-dependent manner, which are transferred via CD36 to sustain cancer cell growth. Additionally, CAF-derived LPC is converted to mitogenic LPA, promoting cellular proliferation. This process is further intensified by Treg metabolic reprogramming through CD36-PPARβ signaling and FoxP3-mediated optimization of OXPHOS. In parallel, circuits evading ferroptosis are characterized by the activation of NRF2, which transactivates SLC7A11 to enhance cystine uptake, thereby reactivating GPX4 and facilitating the clearance of lipid peroxides. Collectively, these lipid-driven defense mechanisms, comprising genotoxic stress mitigation, bioenergetic stability, immunosuppressive niche formation, and reprogramming of cell death pathways, form a dynamic resistance architecture that necessitates combinatorial therapeutic strategies to optimize radiotherapy outcomes. Created with BioRender.com.

Consequently, a thorough investigation into how tumors utilize lipid metabolic pathways to establish both intrinsic and acquired radioresistance, while concurrently shaping an immunosuppressive TME, is crucial to overcoming current limitations in radiotherapy and to devising precise combination therapies based on lipid metabolic intervention. This section will systematically elaborate on the lipid metabolism-driven mechanisms underlying radioresistance. **Figure 5** illustrates the integration of these radioresistance defense mechanisms.

### Innate protective mechanisms of lipid metabolic reprogramming in primary radioresistance

The intrinsic lipid metabolic processes that contribute to radioresistance can be hierarchically organized into core and supporting mechanisms. The core mechanisms primarily involve sustained FAO for energy production and cholesterol-mediated membrane stabilization, both of which directly maintain cellular survival under radiation stress. These are further supported by auxiliary processes, including enhanced *de novo* lipogenesis, which provides substrates for membrane synthesis and signaling, as well as LD accumulation, which facilitates metabolic buffering and the mobilization of repair resources. Intrinsic lipid metabolic reprogramming in tumor cells constitutes a pivotal factor contributing to radioresistance. A key characteristic that differentiates malignant cells from normal cells is their persistent activation of biosynthetic pathways to satisfy elevated metabolic demands, even in the presence of exogenous lipids 34,264-266. Critical rate-limiting enzymes involved in fatty acid synthesis, such as ACLY 267,268, Acyl-CoA Synthetase Short-Chain Family Member 2 (ACSS2) 269,270, ACC 271,272, FASN 273,274, and SCD 275,276, are frequently overexpressed in various tumor types, thereby promoting tumor proliferation, invasion, and metastasis. In radioresistant cells, *de novo* fatty acid synthesis is significantly upregulated, which supports increased production of membrane PLs and lipid signaling mediators 277. Clinically, elevated ACLY expression is associated with decreased overall survival in patients with head and neck squamous cell carcinoma undergoing radiotherapy (clinical observation). Mechanistically, the inhibition of ACLY compromises the capacity for DNA damage repair and enhances the radiosensitivity of tumor cells (cellular models) 278-280. In a similar vein, the expression and activity of FASN are upregulated in radioresistant nasopharyngeal and pancreatic carcinomas, correlating with a poor prognosis (clinical observation) 260,281,282. In the context of prostate cancer, FASN overexpression leads to the upregulation of the androgen receptor via the Akt/NF-κB signaling pathway, induces cell cycle arrest, enhances DNA repair mechanisms, and ultimately contributes to radioresistance (cellular models) 283-285. Furthermore, FASN facilitates radioresistance in nasopharyngeal carcinoma through FZD10-mediated signaling pathways 260. Importantly, the inhibition of SCD in radioresistant cells induces the compensatory secretion of the atypical unsaturated fatty acid sapienate via FADS2, a metabolic pathway typically active in sebaceous glands, thereby underscoring the ability of tumor cells to sustain survival through adaptive metabolic rewiring (cellular models) 260.

FAO constitutes a vital energy supply pathway in radioresistant tumors. Cells exhibiting radioresistance often demonstrate increased FAO activity and overexpression of related enzymes 261,286,287. In breast cancer models, the expression of CD47 is influenced by FAO metabolites, which facilitates the evasion of macrophage-mediated phagocytosis (cellular models) 288,289. The combination of FAO inhibitors, such as the CPT1 inhibitor etomoxir, with anti-CD47 antibodies has been shown to enhance tumor control in cases of glioblastoma recurrence following radiotherapy (radiosensitization experiments) 286. Long-chain ACSL, a member of the ACSL enzyme family, plays a role in enhancing DNA damage repair and inhibiting apoptosis by regulating FOXM1, thereby contributing to radioresistance (cellular models) 290. In nasopharyngeal carcinoma, both FAO activity and the expression of its rate-limiting enzyme CPT1A are elevated in radioresistant cells, correlating with reduced survival rates post-radiotherapy (clinical observation) 13. Mechanistically, CPT1A facilitates fatty acid transport through its interaction with Rab14, which mitigates radiation-induced lipid peroxidation and promotes resistance (cellular models) 13. Furthermore, the PGC1α/CEBPB/CPT1A signaling axis enhances radioresistance in nasopharyngeal carcinoma through the activation of FAO (cellular models) 291. In the context of radioresistant breast cancer and breast cancer stem cells, inhibition of FAO, achieved either through CRISPR-mediated knockout of CPT1A/CPT2 or via pharmacological means, attenuates radiation-induced ERK activation, reduces invasive potential, and diminishes radioresistance (radiosensitization experiments) 261.

Cholesterol metabolic remodeling plays a crucial role in establishing a radioprotective barrier within tumor cells. Transcriptomic analyses consistently link genes involved in cholesterol synthesis, such as FDPS, ACAT2, AG2, and SLC12A2, with radioresistance across various cancer cell lines (clinical observation) 292. The enzyme HMGCR, which serves as the rate-limiting step in cholesterol biosynthesis, is found to be upregulated in gastric cancer, glioblastoma, and prostate cancer (clinical observation) 222. The combination of the HMGCR inhibitor pitavastatin with radiotherapy has been shown to increase the persistence of DNA double-strand breaks (DSBs), induce cellular senescence, and enhance the efficacy of radiotherapy in resistant melanoma (radiosensitization experiments) 222. Farnesyl pyrophosphate synthase (FDPS), a key branchpoint enzyme in cholesterol synthesis, is overexpressed in pancreatic ductal adenocarcinoma and is associated with poor response to radiotherapy and reduced survival rates (clinical observation) 292,293. Inhibition of FDPS using zoledronic acid has been demonstrated to radiosensitize tumors by disrupting Rac1 and Rho isoprenylation, thereby enhancing systemic immune activation (radiosensitization experiments) 293. Additionally, cellular cholesterol levels influence membrane protein function; for example, ABCG2 is implicated in mediating cholesterol-induced chemoresistance in non-small cell lung cancer. Statins have been shown to downregulate ABCG2 by decreasing cholesterol levels, thereby mitigating chemoresistance (cellular models) 294. Additionally, cholesterol acts synergistically with saturated fatty acids to enhance the malignancy of prostate cancer (cellular models) 295. As derivatives of cholesterol, oxysterols exert an indirect influence on DNA repair mechanisms through the activation of LXR. In the context of colon cancer, activation of LXR by oxysterols leads to the suppression of Ku70 expression, a crucial protein involved in the non-homologous end joining (NHEJ) repair pathway, thereby reducing radiosensitivity (cellular models) 296.

LDs, which serve as the primary storage sites for TAGs and cholesterol, play a crucial role in conferring resistance to oxidative stress through dynamic lipid storage mechanisms. Inhibition of diacylglycerol O-acyltransferase (DGAT) 2, an essential enzyme involved in LD biogenesis, leads to a reduction in LD content, suppression of cell migration, and increased sensitivity of MCF7 breast cancer cells to radiotherapy (radiosensitization experiments) 8. The abundance of LDs is intricately linked to iron homeostasis, particularly through the expression of ferritin heavy chain (FTH1). In breast and lung cancer cells, silencing of FTH1 results in decreased LD content and the induction of radioresistance, whereas overexpression of FTH1 or iron chelation using deferoxamine can reverse this phenotype (cellular models) 297. The sequestration of toxic fatty acids mediated by DGAT1/2 provides protection against ferroptosis, which is induced by lipid peroxidation (cellular models) 298,299. Furthermore, LDs have the capability to release fatty acids via autophagic pathways, thereby supporting rapid repair processes following radiotherapy (cellular models) 300-302.

### Adaptation in lipid metabolism induced by radiotherapy facilitates the acquisition of radioresistance

Radiotherapy induces dynamic alterations in tumor lipid metabolism, facilitating the development of adaptive radioresistance. In nasopharyngeal carcinoma cells, exposure to radiation markedly increases the expression of CPT1A. This upregulation enhances the transport of fatty acids from LDs to mitochondria through interaction with Rab14, thereby mitigating radiation-induced lipotoxicity and promoting FAO for ATP production. This process supports energy metabolism and contributes to treatment resistance. Clinical studies have demonstrated a significant correlation between elevated CPT1A expression and decreased survival rates in nasopharyngeal carcinoma patients post-radiotherapy (clinical observation) 13. Furthermore, radiotherapy activates the FASN/PGC1α axis, leading to increased *de novo* fatty acid synthesis. In radioresistant prostate cancer cells, overexpression of FASN upregulates the androgen receptor via the Akt/NF-κB pathway, induces cell cycle arrest, and enhances DNA repair capabilities (cellular models) 283. Additionally, the transcriptional coactivator PGC1α collaborates with CEBPB to further promote CPT1A expression, thereby amplifying FAO flux (cellular models) 291.

Membrane phospholipid remodeling constitutes a pivotal survival mechanism employed by tumor cells in response to radiotherapy. In radioresistant breast cancer cells, there is a significant upregulation in the synthesis of PC and PE. This increase, in conjunction with elevated GSH levels, enhances the scavenging of ROS and mitigates radiation-induced oxidative damage. The inhibition of GSH synthesis through buthionine sulfoximine leads to increased ROS production and induces apoptosis, thereby counteracting radioresistance (radiosensitization experiments) 303. Additionally, enhanced cardiolipin synthesis plays a crucial role in maintaining mitochondrial membrane stability. In hepatocellular carcinoma cells, the upregulation of cardiolipin synthesis following radiotherapy inhibits radiation-induced cytochrome C release, thereby preventing the initiation of apoptosis (cellular models) 16.

Cholesterol metabolic reprogramming plays a significant role in adaptive radioresistance. In colorectal cancer cell lines HCT-8 and HT-29, radiotherapy activates the SREBP1/FASN signaling pathway, leading to increased cholesterol biosynthesis, maintenance of cancer stem cell characteristics, and enhanced proliferation. Silencing of SREBP1 or pharmacological inhibition of FASN, for instance with TVB-2640, has been shown to increase radiosensitivity (radiosensitization experiments) 304. Cholesterol enrichment within lipid rafts facilitates the aggregation of DNA repair proteins. The radioresistant head and neck squamous cell carcinoma cell line SQ20B demonstrates substantially higher lipid raft cholesterol content compared to the radiosensitive line SCC61; depletion of cholesterol, such as through methyl-β-cyclodextrin (MβCD) treatment, results in increased ROS production and apoptosis following irradiation (radiosensitization experiments) 305. Additionally, cholesterol mitigates intracellular ROS accumulation by activating efflux transporters like ABCG2 (cellular models) 294.

### TME lipid metabolic coupling in radioresistance

Irradiation reshapes not only tumor-cell lipid metabolism but also lipid exchange and signaling within the TME. Cancer cells, CAFs, adipocytes, and macrophages can exchange FFAs, cholesterol, LPC, and lipid rich vesicles, thereby supplying substrates for membrane repair, energy production, and adaptive survival after IR. At the same time, increased lipid uptake by tumor and stromal cells may intensify nutrient competition and impair antitumor immunity, particularly by promoting lipotoxic dysfunction or exhaustion of CD8+ T cells. Bioactive lipid mediators, including PGE2, LPA, oxysterols, and S1P, further coordinate inflammatory, immunosuppressive, and survival signaling within the irradiated microenvironment. In addition, exosomes can transfer non-coding RNAs and lipid metabolic enzymes between tumor and stromal compartments, allowing adaptive resistance signals to spread after irradiation. Through these interconnected processes, lipid metabolism links tumor-cell intrinsic radioprotection with immune escape and stromal support.

The metabolic reprogramming of TAMs constitutes a pivotal mechanism in the facilitation of immunosuppression. Fatty acids secreted by cancer cells are internalized by TAMs through the CD36 receptor, leading to enhanced FAO and OXPHOS. The oxidative stress induced by FAO inhibits SHP1, promotes the phosphorylation of STAT6, and increases the expression of arginase-1 (Arg1), thereby promoting the polarization of TAMs towards a pro-tumor M2 phenotype. The pharmacological inhibition of lipid uptake or metabolism in TAMs can facilitate their repolarization towards the anti-tumor M1 phenotype (radiosensitization experiments) 306. Furthermore, interleukin-1 beta (IL-1β) derived from cancer cells induces the expression of the Marco receptor in TAMs, thereby augmenting lipid uptake, while CCL6 secreted by TAMs enhances the migratory capacity of cancer cells (cellular models) 307.

The effector function of T cells is markedly compromised by lipid accumulation within the TME. Post-radiotherapy, the TME exhibits elevated concentrations of FFAs, which facilitate lipotoxicity-induced exhaustion of CD8⁺ T cells via CD36-mediated lipid uptake. This state of exhaustion is characterized by the upregulation of immune checkpoint molecules, such as PD-1, TIM-3, and LAG-3 (cellular models) 308. In the context of breast cancer, FFAs released from tumor tissues directly inhibit the cytotoxic activity of CD8⁺ T cells (cellular models) 309. Additionally, cholesterol accumulation within T cells triggers ER stress, resulting in heightened expression of immune checkpoints and a consequent reduction in anti-tumor efficacy (cellular models) 308.

Tregs employ lipid metabolism to sustain their immunosuppressive function. Cancer-associated adipocytes (CAAs) provide fatty acids to Tregs through CD36, while CD36-PPARβ signaling orchestrates the metabolic adaptation of Tregs, promoting their accumulation and functional persistence within tumor environments (cellular models) 310. The transcription factor FoxP3 strengthens Treg identity by downregulating Myc expression and glycolysis, thus favoring fatty acid synthesis and OXPHOS (cellular models) 311. Additionally, tumor-derived COX-2/PGE2 signaling can augment Treg activity by inducing FoxP3 expression (cellular models) 312.

In addition to its well-documented roles in immune suppression and ferroptosis resistance, lipid metabolic reprogramming plays a significant role in therapy resistance through various mechanisms. Notably, the remodeling of phospholipid membranes results in a denser and less fluid membrane structure. This alteration in biophysical properties can enhance the plasma membrane's barrier function, thereby potentially reducing the penetration and intracellular accumulation of certain cytotoxic drugs 62,313. Moreover, compelling preclinical evidence indicates that targeting pivotal nodes in lipid metabolism can effectively overcome this resistance. Inhibition of key enzymes, such as ACLY, FASN, or SCD, has been shown across various cancer models to significantly sensitize tumor cells to chemotherapy, radiotherapy, and targeted therapies (radiosensitization experiments) 314-317. Together, these findings indicate that lipid metabolic reprogramming contributes to therapy resistance both by limiting drug delivery and by creating targetable metabolic dependencies.

### Lipid crosstalk between stromal and cancer cells promotes adaptive radioresistance

CAFs play a pivotal role as lipid contributors within the TME. In the context of colorectal cancer, the expression of FASN is elevated in CAFs, leading to the secretion of FFAs that are subsequently absorbed by cancer cells via the CD36 receptor, thereby enhancing radioresistance. This mechanism can be mitigated by either silencing FASN in CAFs or inhibiting CD36 in cancer cells (radiosensitization experiments) 318. In pancreatic cancer, activated pancreatic stellate cells (PSCs) generate LPC, which is taken up by cancer cells for the synthesis of PC or its conversion into LPA. LPA functions as a mitogenic signaling molecule, promoting cancer cell proliferation in response to IR (cellular models) 319.

The exosome-mediated transfer of lipid metabolic enzymes post-radiotherapy has emerged as a novel mechanism for the dissemination of resistance phenotypes. Tumor cell-derived exosomes transport non-coding RNAs, such as miRNA, which activate the PI3K/AKT/mTOR pathway to enhance fatty acid synthesis (cellular models) 320-323. In glioblastoma, exosomes enriched with circATP8B4 following radiotherapy augment radioresistance in recipient cells by acting as a sponge for miR-766 (cellular models) 324. Furthermore, the lncRNA AGAP2-AS1 contained in M2 macrophage-derived exosomes reduces radiotherapy sensitivity in lung cancer by modulating the miR-296/NOTCH2 axis (cellular models) 325.

### Primary resistance mechanisms include evasion of ferroptosis and metabolic adaptability

Radiotherapy-resistant tumors maintain their survival by enhancing mechanisms that defend against ferroptosis. Radiation exposure can lead to the upregulation of SLC7A11 expression, which facilitates cystine uptake and promotes the synthesis of GSH. This process subsequently activates GPX4, which detoxifies lipid peroxides and inhibits ferroptosis (cellular models) 326-328. In glioma cells that exhibit radioresistance, mutations in KEAP1 result in the constitutive activation of NRF2. This activation increases SLC7A11 transcription through antioxidant response elements (ARE), thereby contributing to enhanced radioresistance (cellular models) 3. Furthermore, NRF2 activates the FSP1-CoQ10 antioxidant pathway: ferroptosis suppressor protein 1 (FSP1) reduces ubiquinone (CoQ10) to ubiquinol, which scavenges lipid peroxides independently of GPX4 and effectively inhibits ferroptosis in KEAP1-mutant lung cancer (cellular models) 329-331. Although the activation of p53 following radiotherapy can suppress SLC7A11 expression and promote ferroptosis, tumors deficient in p53 can evade ferroptosis by maintaining high levels of SLC7A11 expression (cellular models) 332.

Resistance to radiation-induced ferroptosis arises from coordinated mechanisms that limit lipid peroxidation and iron-dependent oxidative damage. A central axis is NRF2-KEAP1 activation, which upregulates SLC7A11 and GPX4 to enhance glutathione-dependent antioxidant capacity 3,215,216. In parallel, iron availability is restricted through FTH1-mediated sequestration and Prominin2-driven efflux, further suppressing ferroptotic susceptibility 333,334. Lipid remodeling also contributes, as reduced ACSL4 limits PUFA-containing phospholipids, whereas ACSL3-driven MUFA synthesis stabilizes membranes against oxidation 335,336. Importantly, the contribution of ferroptosis is highly context-dependent. Tumors with high PUFA content or hypoxia-driven lipid remodeling tend to be more sensitive, whereas NRF2-activated or KEAP1-mutant tumors exhibit intrinsic resistance 337-340. Together, these findings indicate that ferroptosis in radiotherapy is not universal but determined by tumor-specific redox balance, lipid composition, and genetic background.

Metabolic plasticity constitutes a fundamental mechanism by which tumors adapt to the stress induced by radiotherapy. Glycolipid metabolic coupling is pivotal in the post-irradiation microenvironment, where radiotherapy enhances glycolytic flux in tumor cells, resulting in lactate production. This lactate activates HIF-1α via the G protein-coupled receptor GPR81, subsequently upregulating FASN, thereby promoting lipid accumulation (cellular models) 36,341,342. In the context of pancreatic cancer, lactate stimulates MDSCs through the GPR81/mTOR/HIF-1α/STAT3 signaling pathway post-radiotherapy, thereby creating an immunosuppressive environment (cellular models) 341,343.

Additionally, glutamine metabolic reprogramming plays a crucial role in conferring radioresistance. Glutamine synthetase (GS) is overexpressed in hepatocellular and breast carcinomas, facilitating the conversion of glutamate and ammonia into glutamine. This process provides precursors for nucleotide synthesis, including purines and pyrimidines, thereby supporting DNA damage repair (DDR) mechanisms. The silencing of the GS gene compromises the efficiency of homologous recombination repair and enhances radiosensitivity (radiosensitization experiments) 228,344.

## Strategies for Overcoming Radioresistance through the Modulation of Lipid Metabolism

Tumor cells construct an extensive defensive network through intrinsic lipid metabolic reprogramming, adaptive responses induced by radiotherapy, and lipid-mediated interactions within the TME, collectively contributing to the development of radioresistance. As discussed in the preceding section, tumors employ multiple strategies to evade radiation-induced cell death, including the enhancement of *de novo* fatty acid synthesis and oxidation, the remodeling of cholesterol homeostasis, and the dynamic utilization of LDs as storage organelles, all of which contribute to a primary resistance barrier. During radiotherapy, acquired adaptations further bolster resistance through the upregulation of CPT1A, activation of the FASN/PGC1α axis, reconstruction of membrane PLs, and promotion of cholesterol biosynthesis.

Within the TME, alterations in lipid metabolism are critically implicated in immunosuppression. Specifically, the uptake of lipids by TAMs facilitates M2 polarization, while lipotoxicity-induced exhaustion detrimentally affects the function of CD8⁺T cells. Furthermore, the reliance on fatty acids enhances the suppressive activity of Tregs. The transfer of lipids, mediated by CAFs and exosomes, collectively contributes to the establishment of an immunotolerant niche. Central to these processes are the mechanisms of ferroptosis evasion and metabolic plasticity, which function as integrative hubs for pro-survival and immunosuppressive signaling pathways.

**Figure 6:**
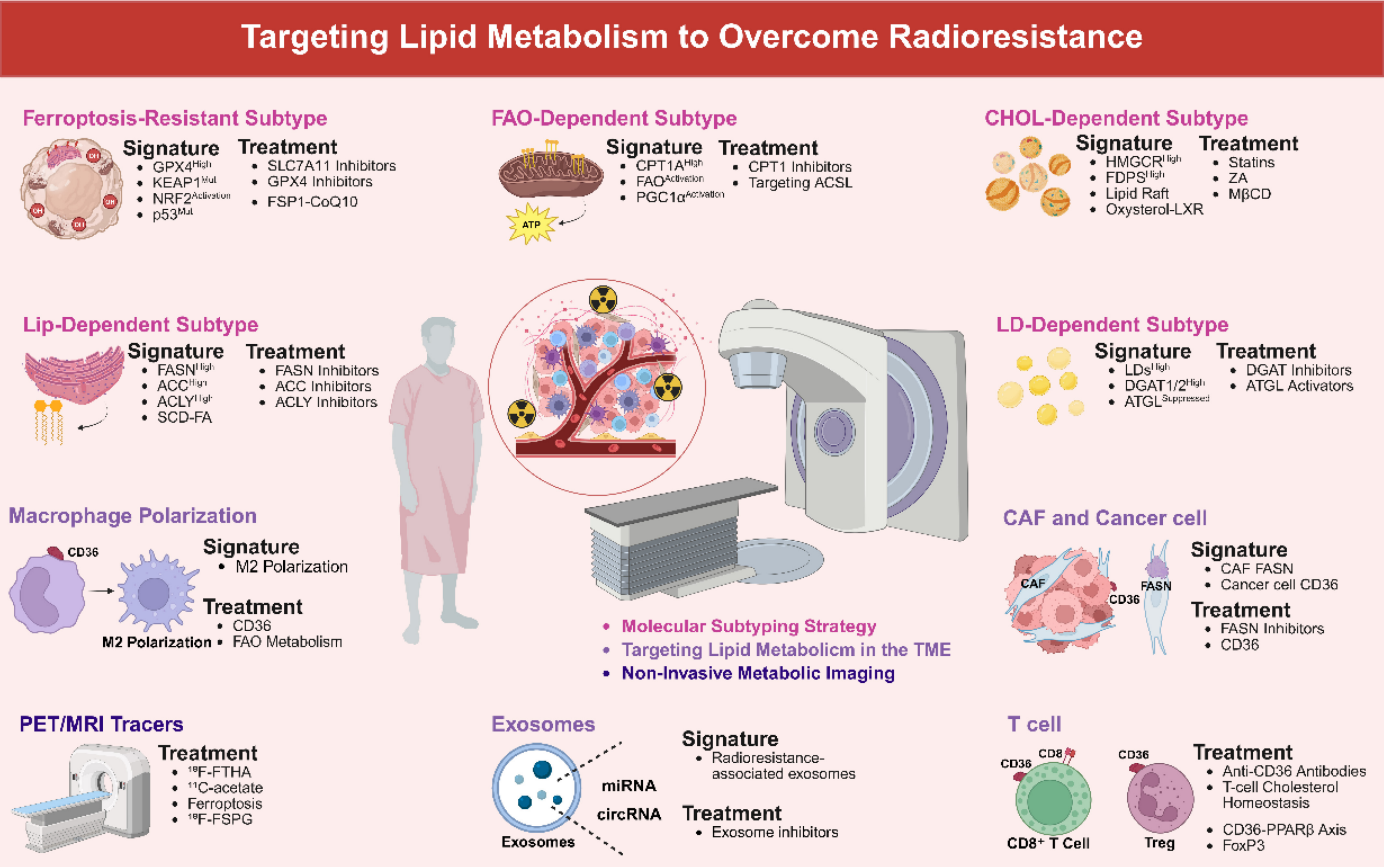
** Therapeutic Exploitation of Lipid Metabolic Vulnerabilities for Precision Radiosensitization.** This graphical abstract outlines three synergistic therapeutic strategies aimed at overcoming radioresistance by targeting tumor lipid metabolism. The approach involves molecular subtyping-guided targeting, which stratifies tumors into specific cohorts based on their metabolic characteristics. For ferroptosis-resistant tumors characterized by high GPX4 expression, active NRF2, and p53 deficiency, treatment involves the use of SLC7A11 inhibitors, such as sulfasalazine analogs, to induce cystine starvation, along with GPX4 catalytic blockers to inhibit peroxide reduction. FAO-dependent malignancies, identified by high CPT1A and PGC1α expression, are targeted with CPT1 inhibitors like etomoxir to block mitochondrial fatty acid import, and ACSL4 inhibitors such as AS-252424 to prevent the esterification of arachidonic acid into ferroptosis-susceptible phospholipids. Lipid synthesis-addicted tumors, marked by elevated levels of FASN, ACLY, and ACC, are addressed with FASN inhibitors to disrupt palmitate-dependent MHC-I lysosomal degradation, thereby enhancing the anti-PD-L1 response, and ACLY/ACC blockers to inhibit NF-κB signaling. Lastly, cholesterol-dependent phenotypes, characterized by high HMGCR and FDPS expression, are targeted with lipophilic statins to impair HMGCR-mediated mevalonate flux, or with zoledronic acid to inhibit FDPS-mediated isoprenylation of Rac1/CDC42, thereby affecting DNA repair mechanisms. In tumors characterized by lipid droplet accumulation, specifically those with high DGAT1/2 and low ATGL expression, the application of DGAT inhibitors effectively redistributes sequestered PUFAs to cellular membranes, thereby restoring sensitivity to ferroptosis. Disruption of TME lipid crosstalk is achieved through the use of CD36 monoclonal antibodies, which inhibit lipid uptake by metastasis-initiating cells, in conjunction with CAF-targeted FASN inhibition, which reduces FFA secretion. This approach concurrently disrupts the exosomal transfer of miRNA and circRNA, which are involved in reprogramming the metabolism of recipient cells. Additionally, CD36 blockade mitigates the ferroptosis of CD8⁺ T cells induced by ω-6 PUFAs, while depletion of CAF-derived palmitate downregulates the expression of the "don't eat me" signal CD47 on cancer cells. The integration of non-invasive theranostic techniques involves the use of 11C-acetate and 18F-FDG dual-tracer PET imaging to differentiate between *de novo* lipogenesis and glycolytic activity. Furthermore, 11C-palmitate dynamic modeling is employed to quantify FAO flux, and124I-labeled GPX4 antibodies with triple coincidence detection are used to assess vulnerability to ferroptosis. This is complemented by liquid biopsy monitoring of exosomal miRNA and circRNA signals that indicate metabolic reprogramming, as well as serum biomarkers such as 4-HNE and 8-iso-PGF2α, which reflect oxidative stress and correlate with 68Ga-FAPI-46 and 18F-FSPG multi-tracer imaging results. This precision framework, when considered collectively, facilitates metabolic subtype-specific radiosensitization, intercepts the stromal-cellular lipid axis, and allows for real-time response verification, thereby overcoming therapeutic resistance. Created with BioRender.com.

Research into these pathways has identified targetable metabolic vulnerabilities, offering a rational foundation for the development of innovative radiosensitization strategies. Future efforts in combination therapies targeting lipid metabolism should focus on three fundamental aspects: personalized interventions informed by metabolic molecular subtyping, reprogramming of the immunosuppressive TME, and the advancement of dynamic monitoring technologies to observe metabolic adaptation in real time. Ultimately, these strategies aim to overcome therapeutic resistance and enhance clinical outcomes in radiotherapy. **Figure 6** illustrates precision radiosensitization strategies that exploit lipid vulnerabilities.

Accordingly, the therapeutic framework in this section is organized by resistance mechanism rather than by drug class: ferroptosis evasion is targeted by SLC7A11/GPX4/FSP1 inhibitors, FAO-dependent adaptation by CPT1 blockade, lipogenic membrane repair by FASN/ACC/ACLY inhibition, cholesterol raft-mediated repair by statins or FDPS inhibitors, and LD-mediated buffering by DGAT/ATGL-directed interventions.

### Developing therapeutic strategies informed by molecular classification

Enhancing the efficacy of radiotherapy requires precise stratification based on the metabolic molecular profiles of tumors. Tumors resistant to ferroptosis often exhibit high GPX4 expression, KEAP1 mutations leading to constitutive NRF2 activation, and p53 dysfunction 345-348. To counteract ferroptosis evasion driven by these mechanisms, these tumors may be particularly amenable to combination therapies that include ferroptosis-inducing agents. Inhibitors of SLC7A11, such as sulfasalazine and its derivatives, impede GSH synthesis by obstructing cystine uptake, thereby disrupting the SLC7A11-GSH-GPX4 antioxidant axis 183,349,350. Small-molecule inhibitors that directly target the catalytic activity of GPX4 eliminate its ability to reduce lipid peroxides, specifically disabling the GPX4-mediated ferroptosis defense 183,191,192,351. Additionally, novel FSP1 inhibitors, which interfere with the FSP1-CoQ10 antioxidant axis by inhibiting FSP1-mediated CoQ reduction at the plasma membrane, target the NRF2/FSP1 backup system 352,353.

Tumors reliant on FAO demonstrate elevated expression of CPT1A, increased FAO flux, and activation of peroxisome proliferator-activated receptor gamma coactivator 1-alpha (PGC1α) signaling pathways. To disrupt radioresistance sustained by FAO-dependent energy supply and redox balance, inhibitors of CPT1, such as etomoxir, impede the mitochondrial import of long-chain fatty acids. In glioblastoma models that exhibit a metabolic shift from glycolysis to FAO following radiotherapy, etomoxir effectively inhibits CD47-mediated immune evasion and enhances the phagocytic activity of macrophages by crippling FAO-driven ATP production 286. Furthermore, targeting the ACSL long-chain family can influence sensitivity to ferroptosis. Specifically, ACSL4 facilitates the incorporation of AA into membrane PLs, creating substrates for ferroptosis; its inhibitor, AS-252424, suppresses lipid peroxidation and enhances radiosensitivity by inhibiting AA-CoA synthesis to counteract membrane PUFA peroxidation 47. Importantly, ACSL3 contributes to membrane stability through the metabolism of saturated fatty acids, and its inhibition may mitigate the pro-ferroptotic effects resulting from the loss of ACSL4 47. The development of subtype-selective inhibitors is crucial for achieving precise therapeutic targeting.

Tumors reliant on lipid synthesis exhibit heightened expression of FASN, ACC, and ACLY, in conjunction with SCD-mediated fatty acid desaturation. To impair *de novo* lipogenesis that supports membrane repair and DDR-mediated radioresistance, inhibitors of FASN attenuate the palmitoylation of MHC-I by curtailing palmitate synthesis, thereby averting its lysosomal degradation and markedly enhancing antigen presentation efficiency in hepatocellular carcinoma 354. In the context of breast cancer, proton pump inhibitors downregulate poly (ADP-ribose) polymerase 1 (PARP1) expression and impair the non-homologous end joining repair mechanism by inhibiting FASN thioesterase activity, thereby blocking FASN-facilitated DNA repair 14. Furthermore, allosteric inhibitors of ACC and blockers of ACLY can synergistically impede *de novo* lipogenesis, disrupt membrane integrity, and inhibit acetyl-CoA-dependent NF-κB signaling 14,286.

Preclinical investigations have elucidated that targeting critical nodes within lipid metabolism can enhance the efficacy of radiotherapy. Specifically, ACSL4 inhibitors have been shown to reduce the esterification of arachidonic acid and adrenic acid into phospholipids, thereby mitigating radiation-induced lipid peroxidation and ferroptosis 355. Additionally, inhibitors of the Xc⁻ system, such as erastin, deplete glutathione by obstructing cystine uptake, which synergizes with radiotherapy to induce ferroptosis 356. Furthermore, GPX4 inhibitors like RSL3 directly impair the repair of phospholipid hydroperoxides, resulting in a 78% tumor regression in murine models when used in conjunction with radiation 3. The FASN inhibitor TVB-2640 disrupts *de novo* lipogenesis and DNA repair mechanisms, thereby sensitizing tumors to radiotherapy in preclinical models 283. Clinically, the Phase II trial NCT03808558, which evaluates the combination of TVB-2640 and radiotherapy for FASN-overexpressing solid tumors, has reported preliminary disease control in 40% of evaluable patients, although dose-limiting ocular toxicity remains a significant concern 357. The alkylphospholipid ether analog CLR127 has demonstrated radiosensitizing effects in preclinical studies 357**.** However, it has not progressed to large-scale clinical trials. Currently, there are no Phase III trials investigating the combination of ACSL4 or GPX4 inhibitors with radiotherapy. Critical challenges in this area include optimizing dosage, validating biomarkers, and managing tissue-specific toxicity.

Cholesterol-dependent tumors exhibit dysregulated activation of the MVA pathway, characterized by increased expression of HMGCR and FDPS, along with cholesterol accumulation in lipid rafts 358,359. Radiotherapy further stimulates the MVA pathway through SREBP2-mediated transcriptional activation, leading to enhanced cholesterol synthesis and increased FDPS expression 360,361. Lipophilic statin administration, aimed at influencing cholesterol raft-mediated DNA repair and membrane rigidification, has been correlated with enhanced overall and tumor-specific survival rates in patients with nasopharyngeal carcinoma undergoing concurrent chemoradiotherapy 362. Nonetheless, the clinical application of high-dose statins is constrained by the risk of muscle toxicity 363-365. Alternative strategies involve the use of zoledronic acid and cholesterol-depleting agents. Zoledronic acid specifically inhibits FDPS, thereby preventing the prenylation of Rac1/CDC42 and disrupting the ATM-Rad50 DNA repair complex to radiosensitize tumors 293,366,367. Among cholesterol-depleting agents, MβCD disrupts lipid raft microdomains and interferes with EGFR/PI3K/AKT signaling 368, fluidizing membranes to enhance ferroptosis 369.

Tumors that accumulate LDs utilize these structures as essential energy reservoirs to adapt to the stress induced by radiotherapy. This adaptation is facilitated by the upregulation of DGAT1/2 expression and the suppression of ATGL activity. This LD-mediated sequestration of toxic PUFAs and oxidative stress buffering represents a targetable resistance mechanism. Following radiation-induced cell cycle arrest, there is an increased reliance on LDs due to DGAT-mediated sequestration of PUFAs. These tumor cells continuously absorb extracellular lipids, with DGAT upregulation promoting the storage of PUFAs as TAG within LDs. This process reduces the PUFAs content in membrane PLs, thereby mitigating lipid peroxidation and ferroptosis 298. This mechanism is particularly pronounced in antimetabolite-resistant cell lines. DGAT inhibition directly counteracts this protective mechanism by facilitating the redistribution of PUFAs to membrane PLs, thereby restoring sensitivity to ferroptosis inducers such as RSL3 298. Furthermore, hypoxic conditions within the TME lead to the expression of hypoxia-inducible gene 2 (HIG-2), which inhibits ATGL activity through the PPARγ-G0S2 pathway, thereby preventing LDs hydrolysis 370. Importantly, the function of ATGL is context-dependent. In breast cancer, elevated ATGL expression promotes FAO and enhances metastatic potential 371, whereas in prostate cancer, reduced ATGL expression is associated with disease progression 370. Context-specific modulation of ATGL disrupts LD-dependent stress adaptation.

Radiotherapy precision can thus be tailored to tumor metabolic heterogeneity, maximizing the specificity and efficacy of targeted interventions while minimizing ineffective treatment and systemic toxicity.

The clinical efficacy of radiosensitizers targeting lipid metabolism is critically reliant on the optimization of drug administration timing in relation to radiation fractions. Mechanistically, agents designed to enhance IR-induced primary damage, such as SLC7A11/GPX4 inhibitors that amplify ferroptosis, should be administered prior to or concurrently with irradiation. This timing maximizes tumor vulnerability by preconditioning antioxidant depletion during the ROS burst. In contrast, agents that target adaptive resistance mechanisms, which are upregulated following IR, such as CPT1A/FAO, FASN/lipogenesis, and SREBP2/cholesterol pathways, should be administered post-radiation exposure. This approach effectively disrupts repair and survival signaling during the inter-fraction period. Furthermore, pharmacokinetic considerations are crucial, ensuring adequate intratumoral drug levels at the time of IR for sensitizers or sustained target suppression throughout treatment for adaptive response blockers, to achieve successful combination therapy 372-374.

Targeting lipid metabolism to enhance radiosensitivity may also carry a potential risk of sensitizing normal tissues, particularly when therapeutic strategies interfere with lipid droplet-mediated lipid buffering. Lipid droplets are conserved organelles that play essential roles in maintaining cellular lipid homeostasis by sequestering excess free fatty acids and preventing their incorporation into toxic lipid species that can trigger endoplasmic reticulum stress, oxidative damage, and apoptosis 375-378. This protective function is especially important under lipotoxic conditions, such as nutrient deprivation or excess saturated fatty acid exposure, and is also required for lipid remodeling and energy substrate management in normal tissues 379,380. In particular, neuronal homeostasis relies on astrocytic lipid droplets to sequester peroxidation-prone PUFAs 381,382. Therefore, disrupting DGAT-mediated triacylglycerol synthesis to redirect PUFAs into membrane phospholipids and enhance ferroptosis sensitivity in tumors may also compromise membrane stability and increase the susceptibility of normal cells to oxidative stress or radiation-induced damage 383-385. Nevertheless, tumor cells often exhibit greater dependence on lipid metabolic pathways and reduced metabolic flexibility compared with normal cells, which may provide a therapeutic window for selective targeting. In addition, dose optimization, tumor-targeted drug delivery, and patient stratification based on metabolic profiles may help mitigate potential toxicity. Thus, although targeting lipid metabolism represents a promising radiosensitization strategy, its clinical translation requires careful evaluation of tissue-specific lipid dependencies, safety profiles, and therapeutic selectivity.

While these lipid-targeted strategies are mechanistically appealing, their readiness for translation varies significantly across different pathways. Certain approaches are predominantly supported by mechanistic or cellular evidence, whereas others have undergone testing in animal models or patient-derived systems. However, only a few demonstrate clinical or early clinical relevance for patients undergoing radiotherapy. Clinical translation of lipid-targeted radiosensitization remains at an early stage. Although a few studies have combined lipid-related agents with radiotherapy or chemoradiotherapy, such as sulfasalazine with stereotactic radiosurgery and statins with chemoradiotherapy, these trials were generally not designed around biomarker-defined lipid metabolic radioresistance. In contrast, several clinical trials of lipid metabolism-targeting agents, particularly FASN inhibitors and statins, have been conducted in non-radiotherapy settings, providing evidence for drug feasibility, safety, and pharmacodynamic modulation. Therefore, **Table 1** integrates both preclinical radiosensitization evidence and available clinical trial information, while highlighting the need for prospective trials directly combining radiotherapy with lipid-targeted interventions.

### Exploring the modulation of lipid metabolic interactions within the tumor microenvironment

To effectively target lipid metabolic interactions within the TME, it is essential to precisely intervene in the lipid exchange between stromal and cancer cells. Cancer cells facilitate metastatic progression by internalizing fatty acids from the TME through CD36. Cells expressing CD36 that initiate metastasis have been demonstrated to promote metastasis in models of oral 386 and prostate cancers 387. Monoclonal antibodies targeting CD36 on cancer cells can inhibit lipid uptake and significantly reduce metastatic dissemination in preclinical studies 386. Simultaneously, the inhibition of FASN activity in CAFs decreases the concentration of FFAs in the microenvironment, thereby compromising the energy supply to cancer cells and limiting their proliferation 388.

Importantly, radiotherapy activates CAFs through the induction of TGF-β 389, which may further enhance lipid secretion. Consequently, the combination of radiotherapy with concurrent suppression of CAF metabolic activity constitutes a rational therapeutic strategy. Additionally, exosomes derived from cancer cells serve as critical mediators of stromal-cancer cell communication by transferring lipid metabolic regulators 390,391. Targeting these exosomes can disrupt their role in metabolic reprogramming 392, providing a foundation for developing inhibitors against pro-metastatic exosomal pathways 391.

The concurrent targeting of FASN in CAFs and CD36 in cancer cells offers a promising strategy for the integrated regulation of immunometabolism. This approach effectively disrupts lipid transfer and alleviates immunosuppression within the TME. In pancreatic cancer models, the inhibition of CD36 has been shown to decrease the release of ω-6 PUFAs by cancer cells, thereby reducing the induction of ferroptosis in CD8⁺ T cells 393. Furthermore, palmitic acid derived from CAFs enhances CD47 expression on cancer cells through the IL-8/CXCR2 signaling pathway 394, and the inhibition of FASN indirectly diminishes this "don't eat me" signal 395. Consequently, targeting the lipid transfer between stromal and cancer cells not only directly impedes metastasis but also remodels the immune landscape, thereby enhancing the effectiveness of combined radiotherapy and immunotherapy.

### Metabolic imaging

The precision monitoring of therapeutic efficacy can be significantly enhanced by utilizing novel non-invasive metabolic imaging technologies. In the context of dual-tracer PET imaging, the use of ¹¹C-acetate as a tracer for fatty acid synthesis, in conjunction with ¹⁸F-FDG, substantially improves the diagnostic sensitivity for hepatocellular carcinoma. This methodology is based on the mechanistic principle that ¹¹C-acetate captures *de novo* lipogenesis, while ¹⁸F-FDG reflects glycolytic activity, thereby offering complementary insights into the metabolic heterogeneity of tumors 396. For imaging the FAO pathway, ¹¹C-palmitate facilitates the visualization of myocardial fatty acid metabolism via FAO 397; however, its application in tumor imaging necessitates integration with dynamic modeling techniques to effectively differentiate the signal from background noise 398. In the realm of ferroptosis monitoring, ¹²⁴I, which is both a positron and gamma emitter, can be employed to label GPX4 antibodies 399. This allows for the non-invasive quantification of this critical ferroptosis enzyme through triple-coincidence detection 400. Simultaneously, Compton cameras capable of imaging both ¹³¹I (364 keV) and ⁶⁵Zn (1116 keV) present a promising method for the assessment of zinc ion homeostasis 401. The glutamine metabolism tracer system xC^-^ tracer / ^18^F-labeled glutamate analog, alongside other multi-tracer strategies such as the combination of ⁶⁸Ga-FAPI-46 with ¹⁸F-FDG, has demonstrated enhanced tumor detection rates, highlighting the clinical significance of metabolic phenotyping 402.

Additionally, liquid biopsy markers can effectively complement imaging techniques. Tumor-derived exosomal miRNA, released post-radiotherapy, has the potential to alter the FAO capacity of recipient cells 403,404, while exosome-mediated circRNA facilitates lipid metabolic reprogramming 405,406. Both of these factors represent promising targets for dynamic monitoring. Circulating oxidative stress markers, such as 4-HNE and 8-iso-PGF2α, have the potential to enhance image interpretation when analyzed in conjunction with serum metabolite profiles. For example, the uptake of ¹¹C-acetate has been associated with the expression of the lipogenic enzyme FASN 407, while GPX4 levels may be deduced from PET signals using ¹²⁴I-labeled GPX4 antibodies 408. Looking ahead, the integration of SPECT/PET multi-tracer imaging, such as employing ⁹⁹ᵐTc-labeled exosomal non-coding RNA probes alongside ¹⁸F-FDG, combined with liquid biopsy markers (e.g., exosomal miR-208a and GPX4 activity), may facilitate the development of an early-warning system for monitoring therapeutic response 408.

To enhance clinical utility, the strategic timing of advanced metabolic imaging is crucial. Baseline scans are instrumental in establishing metabolic subtypes and reference values. Importantly, early post-treatment imaging, conducted within 1-2 weeks or after a few treatment fractions, is capable of detecting metabolic changes that are predictive of long-term outcomes. This facilitates adaptive radiotherapy, such as treatment intensification for non-responders or de-escalation for exceptional responders, and often surpasses anatomical imaging in effectiveness. Mid- and post-treatment scans are utilized to evaluate the overall response, while follow-up imaging is employed to identify metabolically active recurrences. The optimal timing of these imaging modalities is contingent upon the specific pathway being investigated, tracer kinetics, treatment regimen, and the clinical question at hand, necessitating a multidisciplinary approach for personalized patient management 409,410.

## Challenges

**Figure 7:**
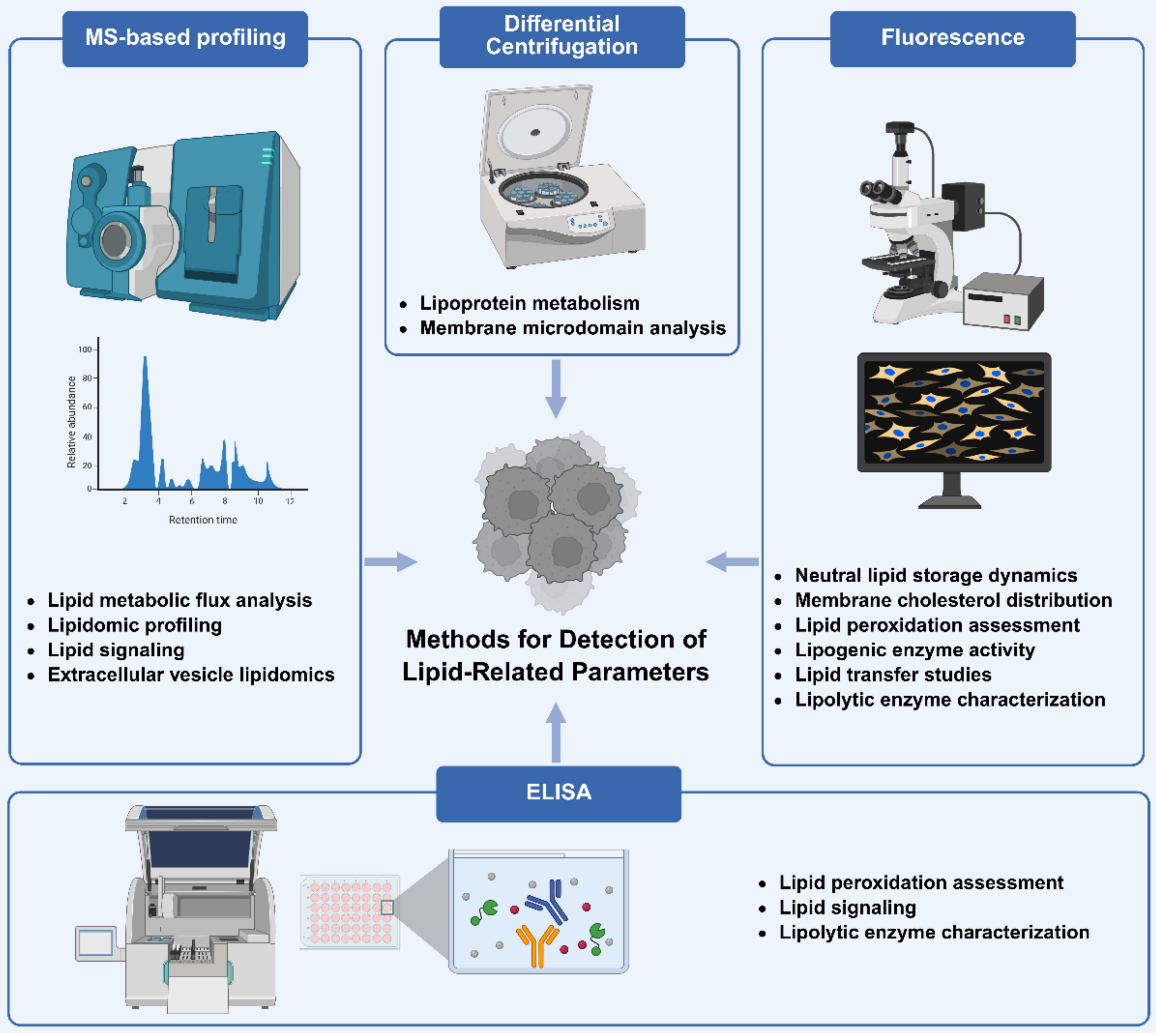
** Methodological Integration for Elucidating the Interplay Between Lipid Metabolism and Radiosensitivity.** This methodologically integrated approach utilizes stable isotope tracing (¹³C/²H) in conjunction with mass spectrometry to examine lipid metabolic flux dynamics through the detection of isotopic enrichment. Concurrently, targeted LC-MS/MS lipidomics facilitates a comprehensive profiling of lipid molecular species, including FFA, TG, PL, CE, Cer, and S1P. Spatial and functional evaluations are conducted using fluorescent BODIPY 493/503 imaging to assess neutral lipid storage dynamics within lipid droplets, Filipin III histochemistry to map membrane cholesterol distribution, and C11-BODIPY spectrofluorometry to quantify lipid peroxidation in irradiated systems. Enzyme-specific investigations employ radiolabeled substrates ([¹⁴C]acetate/[³H]water) to measure the activity of lipogenic enzymes, complemented by fluorescent hydrolysis assays for real-time characterization of lipolytic enzymes such as ATGL, HSL, and MAGL. Systemic lipid metabolism is further dissected through ultracentrifugal fractionation of lipoproteins (VLDL, LDL, HDL) and the isolation of detergent-resistant membranes for lipid raft analysis. Signaling mechanisms are elucidated through the quantification of bioactive lipids, such as LPA, S1P, and PGE2, using enzyme immunoassay and mass spectrometry. This is complemented by the use of fluorescent lipid analogs, such as NBD and BODIPY, to monitor intercellular lipid transfer. The role of extracellular vesicles is assessed through a combination of NanoSight particle counting and liquid chromatography-mass spectrometry lipidomics. This comprehensive framework provides insights into how alterations in lipid synthesis, storage, trafficking, signaling, peroxidation, and vesicular export regulate radiation-induced changes in membrane integrity, DNA damage response, and cell fate decisions. It establishes causal relationships between lipid metabolic networks and cellular radiosensitivity. Created with BioRender.com.

Although lipid metabolism provides multiple opportunities for radiosensitization, the clinical development of lipid-targeted combinations remains at an early stage. Most current evidence comes from cell lines, murine models, organoids, or PDX systems, whereas direct validation in patients receiving radiotherapy is still limited. A major challenge is the redundancy and compensatory flexibility of lipid pathways; for example, inhibition of FAO may induce lipogenic compensation, whereas blockade of *de novo* lipogenesis may increase dependence on exogenous lipid uptake. These adaptive responses highlight the need to define tumor-specific lipid dependencies and to optimize treatment timing in relation to radiation dose and fractionation. Future studies should evaluate lipid-targeted combinations under clinically relevant conditions, including hypoxia, reoxygenation, immune contexture, and radiation schedules that better reflect clinical practice. This methodology for analyzing the interaction between lipids and radiosensitivity is incorporated into **Figure 7** and** Table 2**.

Clinical translation also requires careful consideration of normal-tissue radiobiology. Lipid metabolism is essential for the function and repair of mucosa, liver, skeletal muscle, bone marrow, nervous tissue, and immune cells. Therefore, strategies that enhance lipid peroxidation, inhibit fatty acid synthesis or oxidation, alter cholesterol homeostasis, or impair lipid droplet buffering may also affect normal-tissue tolerance to irradiation. Potential toxicities include hepatotoxicity associated with FASN or CPT1 inhibition, myotoxicity and rhabdomyolysis with high-dose statin-based approaches, hematologic or gastrointestinal injury from ferroptosis-promoting agents, and immune perturbation from interventions targeting CD36 or intercellular lipid transfer 105,411,412. Well-designed clinical studies should incorporate pharmacodynamic lipid markers, normal-tissue toxicity monitoring, and biomarker-enriched enrollment to identify patient populations most likely to benefit from lipid-guided radiosensitization.

Future research endeavors should prioritize the integration of multi-omics data, including genomics, transcriptomics, metabolomics, and radiomics, with artificial intelligence to develop robust predictive models for treatment response. Furthermore, it is imperative to examine how varying radiotherapy dose and fractionation regimens (e.g., hypofractionated versus conventional schedules) differentially affect lipid metabolism within tumors and the TME. This understanding will facilitate the optimization of radiotherapy to enhance its synergy with metabolic interventions. Additionally, expediting the clinical translation of targeted agents that are supported by validated biomarkers, particularly within well-defined molecular subgroups, such as those characterized by KEAP1/NRF2 mutations, p53 deficiency, or CPT1A/FASN overexpression, will be crucial for advancing this promising therapeutic paradigm.

## Conclusions

Tumor lipid metabolic reprogramming significantly affects the response to radiotherapy through various lipid-dependent mechanisms, including radiation-induced lipid peroxidation, membrane remodeling, ferroptosis, bioenergetic stress, DNA damage repair, apoptosis, and immune regulation. The relative significance of these mechanisms is contingent upon factors such as tumor lineage, oncogenic background, baseline lipid composition, oxygenation status, nutrient availability, TME architecture, and the treatment context. Consequently, a comprehensive understanding of the influence of lipid metabolism on radiosensitivity necessitates correlating specific lipid alterations with distinct radiation-associated events, such as oxidative damage, membrane repair, ferroptotic susceptibility, metabolic adaptation, and immune remodeling within the irradiated tumor.

Targeting lipid metabolism presents a promising strategy for overcoming radioresistance, especially when informed by metabolic subtyping and radiation-relevant biomarkers. Future advancements should incorporate strategically timed lipid-targeted interventions alongside TME focused approaches and non-invasive metabolic monitoring. Although many strategies are currently supported primarily by mechanistic or preclinical evidence of radiosensitization, they lay the groundwork for biomarker-driven clinical translation. Conducting prospective studies within molecularly defined patient cohorts, along with a thorough evaluation of normal tissue toxicity and the context of radiotherapy dose-fractionation, will be crucial for advancing lipid-guided radiosensitization in the field of radiation oncology.

Despite ongoing progress, numerous challenges remain, such as the redundancy of metabolic pathways and the tissue-specific toxicities linked to lipid-targeting agents. Future advancements will hinge on the integration of multi-omics data with artificial intelligence to construct predictive models, the optimization of metabolic drug combinations and timing with radiotherapy, and the acceleration of clinical trials within biomarker-defined patient populations. Ultimately, an enhanced understanding of the interaction between lipid metabolism and radiotherapy response will facilitate the development of personalized, metabolism-guided radiotherapy, thereby improving clinical outcomes for cancer patients.

## Figures and Tables

**Table 1:** Translational status of representative lipid-targeted radiosensitization strategies

Strategy	Representative targets/interventions	RT-related rationale	Current evidence	Main translational concern	Clinical trial status and registry link
Ferroptosis enhancement 183,191,192,352,353	SLC7A11/system Xc⁻ inhibitors, GPX4 inhibitors, FSP1 inhibitors	Amplifies IR-induced lipid peroxidation and weakens antioxidant defense	Mechanistic, cellular, and preclinical radiosensitization evidence	Normal-tissue oxidative injury; need biomarkers such as NRF2/KEAP1, SLC7A11, GPX4, and PUFA status	Limited direct RT-combination evidence. Sulfasalazine plus stereotactic radiosurgery has been evaluated in recurrent glioblastoma as a potential tumor-selective radiosensitizer. NCT04205357
FAO inhibition 261,286,291	CPT1A/CPT2 inhibition, etomoxir, PGC1α/CEBPB/CPT1A axis	Limits FAO-dependent ATP production, redox balance, and adaptive survival after IR	Cellular and animal evidence; clinical correlation for CPT1A in NPC	Toxicity and specificity of FAO inhibitors; compensatory lipogenesis	No RT-specific clinical trial identified. Current evidence remains mainly preclinical or correlative; prospective trials combining FAO inhibition with RT are needed.
*De novo* lipogenesis blockade 14,281,354	FASN, ACC, ACLY inhibitors	Impairs membrane repair, lipid-mediated survival signaling, and DNA damage repair support	Cellular and preclinical evidence; limited emerging clinical relevance	Systemic metabolic toxicity; compensation through exogenous lipid uptake or FAO	No dedicated RT-resistance trial identified, but several non-RT oncology trials support clinical feasibility of FASN targeting. TVB-2640/denifanstat has been tested in advanced tumors, KRAS-mutant NSCLC, HER2-positive breast cancer, recurrent high-grade astrocytoma, and resectable cancers. NCT02223247; NCT03808558; NCT03179904; NCT03032484; NCT02980029. Omeprazole has also been studied as a FASN-related strategy in TNBC chemotherapy. NCT02595372
Cholesterol/mevalonate targeting 293,362,368	Statins, FDPS inhibition, zoledronic acid, cholesterol depletion	Disrupts lipid rafts, receptor signaling, prenylation, and DNA repair after IR	Preclinical radiosensitization evidence; clinical observational evidence for statins in RT settings	Dose-limiting myotoxicity; uncertain optimal dose and schedule with RT	Some RT/CRT-combination clinical evidence exists, but not specifically biomarker-selected for lipid-mediated radioresistance. Simvastatin has been combined with capecitabine-based neoadjuvant chemoradiotherapy in rectal cancer. NCT02161822. The SPAR trial also evaluated simvastatin with preoperative chemoradiotherapy in rectal cancer. ACTRN12617001087347. Atorvastatin has been evaluated in high-risk TNBC with standard radiotherapy components. NCT03872388
Lipid droplet disruption 298,370,375	DGAT1/2 inhibition, ATGL/HIG-2 axis modulation	Reduces lipid storage buffering and increases vulnerability to lipid peroxidation	Mainly cellular and preclinical evidence	Therapeutic window unclear; LDs also protect normal tissues from lipotoxic stress	No RT-specific clinical trial identified. DGAT/ATGL-directed strategies remain at the mechanistic and preclinical stage for radiosensitization.
TME lipid crosstalk targeting 386,388,392	CD36 blockade, CAF-FASN inhibition, exosome/lipid-transfer inhibition	Limits stromal lipid supply, immune lipotoxicity, and adaptive resistance signaling	Mostly mechanistic and preclinical evidence	Context-dependent TME effects; potential immune and metabolic toxicity	No RT-specific clinical trial identified. Clinical translation remains exploratory; future trials need biomarker-defined TME lipid-crosstalk endpoints.

**Table 2:** Methodology for analyzing the interaction between lipids and radiosensitivity

Research Objective	Key Technique	Technical Principle	References
Lipid Metabolic Flux Analysis	Stable Isotope Tracing (13C/2H)	Mass spectrometry detection of isotopic enrichment in lipid species after labeled precursor administration	413
Lipidomic Profiling	Targeted LC-MS/MS Lipidomics	Quantitative measurement of lipid molecular species (FFA, TG, PL, CE, Cer, S1P)	414
Neutral Lipid Storage Dynamics	Fluorescent Lipid Droplet Imaging	BODIPY 493/503 staining of neutral lipid cores in intracellular LDs	415,416
Membrane Cholesterol Distribution	Filipin III Histochemistry	Polyene antibiotic binding to unesterified cholesterol in cellular membranes	417
Lipid Peroxidation Assessment	C11-BODIPY Oxidation Assay	Spectrofluorometric detection of oxidized phospholipid membranes	418
Lipogenic Enzyme Activity	Radiolabeled Substrate Conversion Assays	Radioactive-labeled acetate reflects fat metabolic rate	419
Lipoprotein Metabolism	Ultracentrifugal Fractionation	Density-gradient separation of chylomicrons, VLDL, LDL, HDL	420
Lipid Signaling	Bioactive Lipid Quantification (EIA/MS)	Specific measurement of signaling lipids (LPA, S1P, PGE2)	421
Membrane Microdomain Analysis	Detergent-Resistant Membrane Fractionation	Isolation of lipid rafts via sucrose density gradient centrifugation	422
Lipid Transfer Studies	Fluorescent Lipid Analog Tracing	Tracking NBD-labeled or BODIPY-conjugated lipids in live cells	423
Extracellular Vesicle Lipidomics	NanoSight/LC-MS EV Analysis	Combined particle counting and lipidomic profiling of secreted EVs	424
Lipolytic Enzyme Characterization	Fluorescent Substrate Hydrolysis Assays	Real-time measurement of lipase activities (ATGL, HSL, MAGL)	425

## Abbreviations

AA: Arachidonic Acid
ABCG2: ATP-binding cassette sub-family G member 2
ABCA1: ATP-binding cassette transporter A1
ACC: Acetyl-CoA Carboxylase
ACLY: ATP-citrate Lyase
ACO2: Aconitase 2
ACS: Acyl-CoA Synthetase (family)
ACSL: Acyl-CoA Synthetase Long-chain
ACSL4: Acyl-CoA Synthetase Long-chain family member 4
ACSS2: Acyl-CoA Synthetase Short-Chain Family Member 2
AdA: Adrenic Acid
AG2: Apolipoprotein G2
ALOX15: Arachidonate 15-Lipoxygenase
ARE: Antioxidant Response Element
ASAH1: N-acylsphingosine Amidohydrolase 1 (Acid Ceramidase)
ATGL: Adipose Triglyceride Lipase
ATM: Ataxia Telangiectasia Mutated
ATX/ENPP2: Autotaxin/Ectonucleotide Pyrophosphatase/Phosphodiesterase 2
BAD: Bcl-2-associated Death Promoter
BAK: BCL2 Antagonist/Killer 1
BAX: BCL2-associated X Protein
BCL-2: B-cell Lymphoma 2
BCL-XL: B-cell Lymphoma-extra Large
CAAs: Cancer-associated Adipocytes
CAFs: Cancer-associated Fibroblasts
ccRCC: Clear Cell Renal Cell Carcinoma
CD36: Cluster of Differentiation 36
CEBPB: CCAAT/Enhancer-binding Protein Beta
CEs: Cholesteryl Esters
Cer: Ceramide
CerS: Ceramide Synthase
CoQ10: Coenzyme Q10
COX-IV: Cytochrome c Oxidase subunit 4
CPT1A: Carnitine Palmitoyltransferase 1A
CPT2: Carnitine Palmitoyltransferase 2
CYP4A11: Cytochrome P450 Family 4 Subfamily A Member 11
CYP7A1: Cytochrome P450 Family 7 Subfamily A Member 1
CYP27A1: Cytochrome P450 Family 27 Subfamily A Member 1
DAG: Diacylglycerol
DDR: DNA Damage Repair
DGAT1/2: Diacylglycerol O-Acyltransferase 1/2
DHA: Docosahexaenoic Acid
DSBs: DNA Double-Strand Breaks
EGFR: Epidermal Growth Factor Receptor
ERK: Extracellular Signal-Regulated Kinase
FABP3/7: Fatty Acid-Binding Protein 3/7
FAO: Fatty Acid Oxidation
FASN: Fatty Acid Synthase
FADS2: Fatty Acid Desaturase 2
FFAs: Free Fatty Acids
FPP: Farnesyl Pyrophosphate
FDPS: Farnesyl Diphosphate Synthase
FSP1: Ferroptosis Suppressor Protein 1
FTH1: Ferritin Heavy Chain
FZD10: Frizzled class receptor 10
G6PD: Glucose-6-Phosphate Dehydrogenase
GLUL: Glutamate-Ammonia Ligase (Glutamine Synthetase)
GLS: Glutaminase
GPX4: Glutathione Peroxidase 4
GS: Glutamine Synthetase
GSH: Glutathione
GSK3β: Glycogen Synthase Kinase 3 Beta
HER2: Human Epidermal growth factor Receptor 2
HETEs: Hydroxyeicosatetraenoic Acids
HIF-1α: Hypoxia-Inducible Factor 1-alpha
HILPDA: Hypoxia-Inducible Lipid Droplet-Associated
HMGCR: 3-Hydroxy-3-Methylglutaryl-CoA Reductase
HSL: Hormone-Sensitive Lipase
IFNγ: Interferon Gamma
IR: Ionizing Radiation
KEAP1: Kelch-like ECH-Associated Protein 1
LDL: Low-Density Lipoprotein
LDLR: Low-Density Lipoprotein Receptor
LDs: Lipid Droplets
LPA: Lysophosphatidic Acid
LPC: Lysophosphatidylcholine
LPCAT1/2: Lysophosphatidylcholine Acyltransferase 1/2
LPAR1-6: Lysophosphatidic Acid Receptor 1-6
LXR: Liver X Receptor
MAG: Monoacylglycerol
MAPK: Mitogen-Activated Protein Kinase
MβCD: Methyl-β-Cyclodextrin
MDA: Malondialdehyde
MDSCs: Myeloid-Derived Suppressor Cells
MGL: Monoacylglycerol Lipase
MITF: Microphthalmia-Associated Transcription Factor
MOMP: Mitochondrial Outer Membrane Permeabilization
MUFA: Monounsaturated Fatty Acid
mTORC1: Mechanistic Target of Rapamycin Complex 1
NADK1: NAD Kinase 1
NADPH: Nicotinamide Adenine Dinucleotide Phosphate
ND1: NADH:Ubiquinone Oxidoreductase Core Subunit 1
NF-κB: Nuclear Factor kappa-light-chain-enhancer of Activated B cells
NRF2: Nuclear Factor Erythroid 2-Related Factor 2
OXPHOS: Oxidative Phosphorylation
PA: Phosphatidic Acid
PC: Phosphatidylcholine
PDX: Patient-Derived Xenograft
PE: Phosphatidylethanolamine
PE-PUFAs: Phosphatidylethanolamine-conjugated Polyunsaturated Fatty Acids
PGC-1α: Peroxisome Proliferator-Activated Receptor Gamma Coactivator 1-alpha
PI3K: Phosphatidylinositol 3-Kinase
PLA2: Phospholipase A2
PLA2G3/4A: Phospholipase A2 Group III/IV
PLD2: Phospholipase D2
PLIN1-5: Perilipin 1-5
PL-OOH: Phospholipid Hydroperoxides
PPARα/β/γ: Peroxisome Proliferator-Activated Receptor alpha/beta/gamma
PSCs: Pancreatic Stellate Cells
PTEN: Phosphatase and Tensin Homolog
PUFAs: Polyunsaturated Fatty Acids
Rab14: Ras-related protein Rab-14
ROS: Reactive Oxygen Species
SCAP: SREBP Cleavage-Activating Protein
SCD: Stearoyl-CoA Desaturase
S1P: Sphingosine-1-Phosphate
S1PR1-5: Sphingosine-1-Phosphate Receptor 1-5
SGPL1: Sphingosine-1-Phosphate Lyase 1
SHP1: SH2 domain-containing Phosphatase-1
SLC7A11: Solute Carrier Family 7 Member 11
SLC12A2: Solute Carrier Family 12 Member 2
SOAT1: Sterol O-Acyltransferase 1
SPHK1: Sphingosine Kinase 1
SPNS2: Spinster Homolog 2
SRE: Sterol Regulatory Element
SREBP1/2: Sterol Regulatory Element-Binding Protein 1/2
STAT1/3/6: Signal Transducer and Activator of Transcription 1/3/6
TAG: Triacylglycerol
TAMs: Tumor-Associated Macrophages
TCA: Tricarboxylic Acid
TGF-β: Transforming Growth Factor-beta
TFAM: Mitochondrial Transcription Factor A
TIM-3: T-cell Immunoglobulin and Mucin-domain containing-3
TME: Tumor Microenvironment
TNBC: Triple-Negative Breast Cancer
4-HNE: 4-Hydroxynonenal
27-HC: 27-Hydroxycholesterol
